# Exploring the Use of Intracellular Chelation and Non-Iron Metals to Program Ferroptosis for Anticancer Application

**DOI:** 10.3390/inorganics12010026

**Published:** 2024-01-08

**Authors:** Oscar Claudio-Ares, Jeileen Luciano-Rodríguez, Yolmarie L. Del Valle-González, Selene L. Schiavone-Chamorro, Alex J. Pastor, Javier O. Rivera-Reyes, Carmen L. Metzler, Lizandra M. Domínguez-Orona, Brenda Lee Vargas-Pérez, Rachid Skouta, Arthur D. Tinoco

**Affiliations:** 1 Department of Chemistry, University of Puerto Rico, Río Piedras Campus, San Juan, PR 00925, USA;; 2 Department of Science and Technology, Interamerican University of Puerto Rico, Arecibo, PR 00614, USA;; 3 Department of Biology, University of Puerto Rico, Río Piedras Campus, San Juan, PR 00925, USA;; 4 Department of Chemistry, University of Massachusetts Amherst, Amherst, MA 01003-9248, USA;; 5 Department of Biology, University of Massachusetts Amherst, Amherst, MA 01003-9248, USA

**Keywords:** ferroptosis, regulated cell death, chelation, noniron metal complexes

## Abstract

The discovery of regulated cell death (RCD) revolutionized chemotherapy. With caspase-dependent apoptosis initially being thought to be the only form of RCD, many drug development strategies aimed to synthesize compounds that turn on this kind of cell death. While yielding a variety of drugs, this approach is limited, given the acquired resistance of cancers to these drugs and the lack of specificity of the drugs for targeting cancer cells alone. The discovery of non-apoptotic forms of RCD is leading to new avenues for drug design. Evidence shows that ferroptosis, a relatively recently discovered iron-based cell death pathway, has therapeutic potential for anticancer application. Recent studies point to the interrelationship between iron and other essential metals, copper and zinc, and the disturbance of their respective homeostasis as critical to the onset of ferroptosis. Other studies reveal that several coordination complexes of non-iron metals have the capacity to induce ferroptosis. This collective knowledge will be assessed to determine how chelation approaches and coordination chemistry can be engineered to program ferroptosis in chemotherapy.

## 1. Introduction

Approximately 10 million people die annually due to cancer, making it the second most common cause of death globally [1]. Due to the devastating loss of life and increasing numbers of diagnosed people, developing new cancer treatments is one of the most researched topics today. Conventional cancer treatments such as surgery, chemotherapy and radiotherapy are commonly used; however, they can be not only expensive and painful but also most effective when diagnosed early [2]. During the 1960s, a significant breakthrough in chemotherapy research was the discovery of regulated cell death. Commonly referred to as programmed cell death (PCD), regulated cell death (RCD) pertains to a specific type of cell death that can be regulated by a series of specialized molecular mechanisms in pharmacology, molecular biology and genetics [1–3].

**Figure 1.**
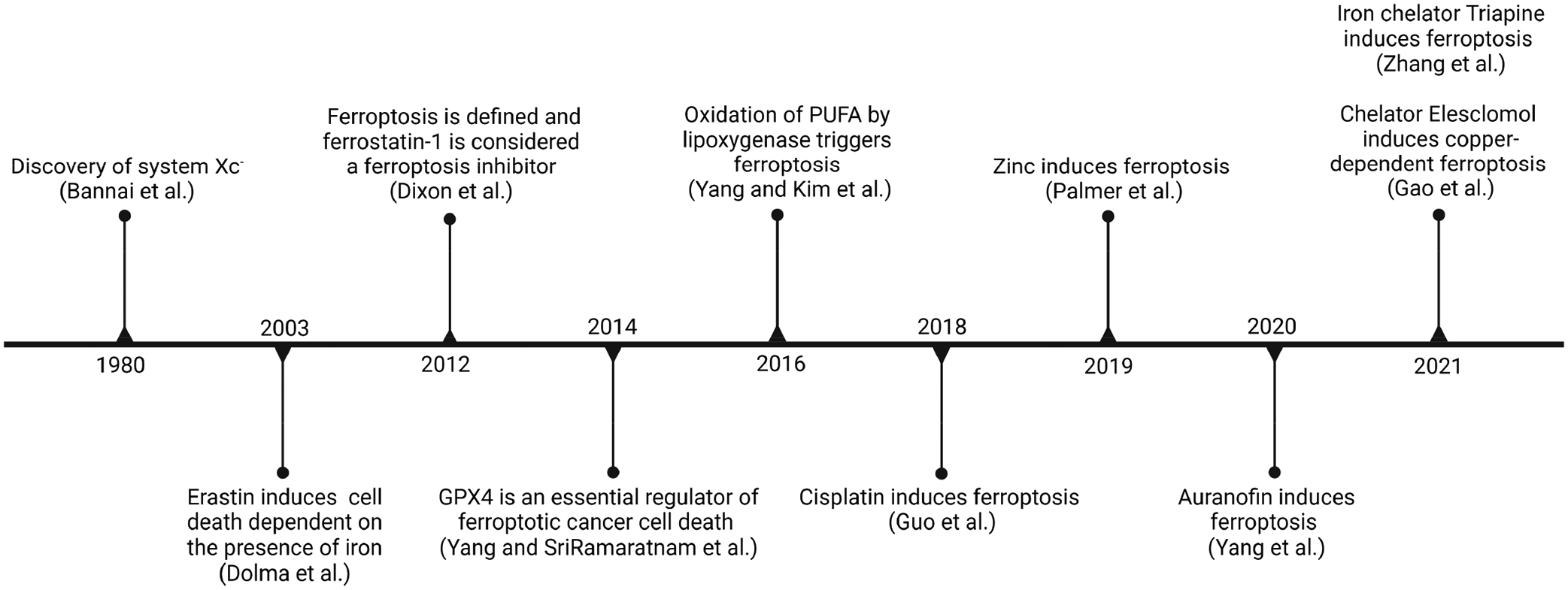
Timeline of ferroptosis-related findings [5–14] (image created using Biorender.com).

Since 1972, caspase-dependent apoptosis was considered the only form of RCD [4]. For this reason, many of the anti-cancer drugs that have been developed target this kind of cell death. Over the years, different cancers have acquired resistance to these treatments, lowering their window of utility. The discovery of new, non-apoptotic forms of RCD is leading the way to the development of drugs targeting these new forms of RCD. In 2012, a non-apoptotic form of RCD, ferroptosis, was documented. This type of cell death is caused by an overproduction of reactive oxygen species (ROS) that forms a buildup of lipid peroxides and is dependent on iron, an essential metal [5]. A timeline of the key developments in ferroptosis-related studies is presented in Figure 1.

**Figure 2.**
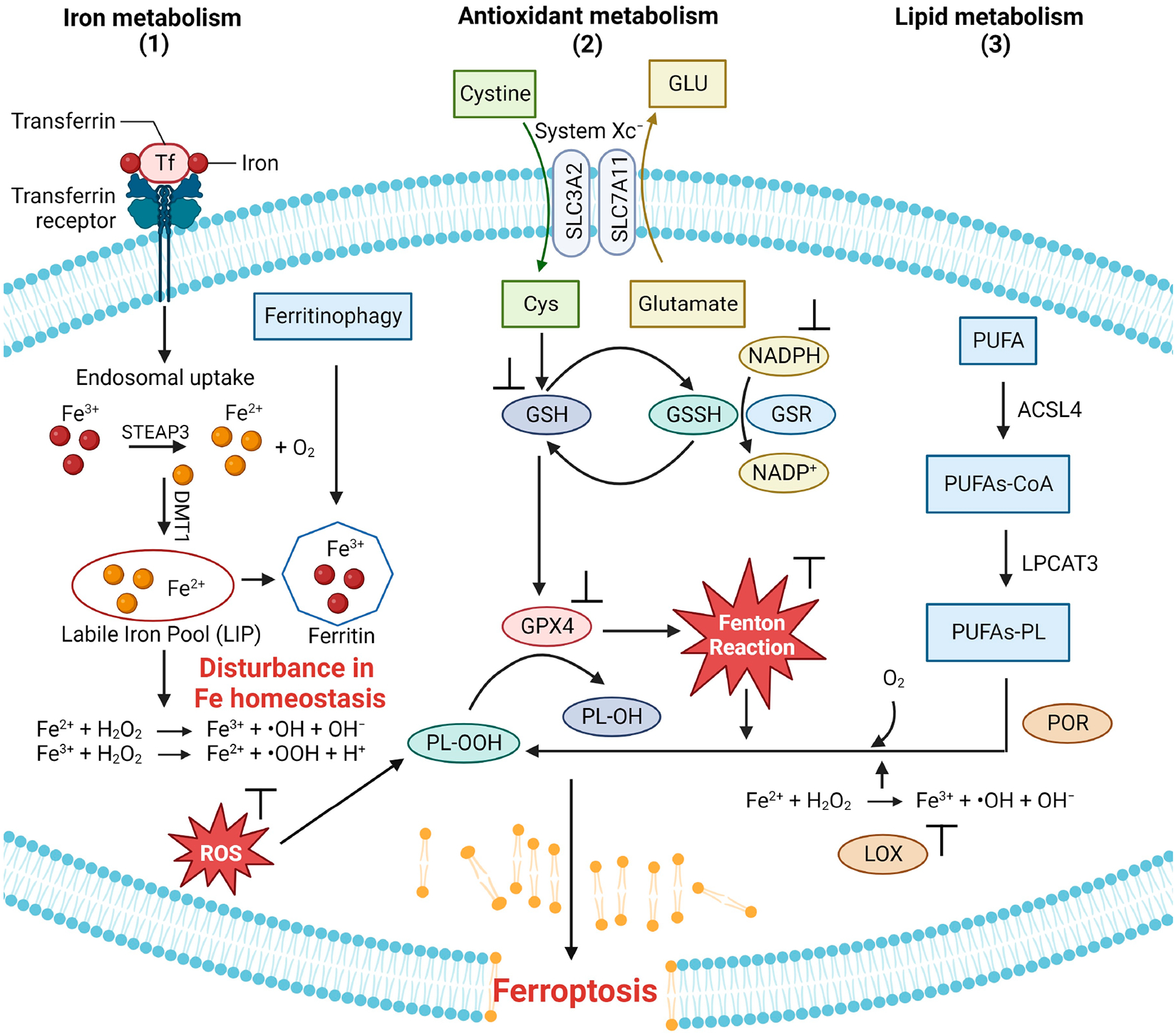
Three major routes of ferroptosis onset related to the metabolism of iron, antioxidants, and lipids (image created using Biorender.com).

Ferroptosis is brought about by three primary mechanisms [15]. Among them, the first is associated with the dysregulation of iron metabolism (Figure 2, route 1). Normally, Fe(III) is delivered to cells throughout the body by the serum transporter transferrin (sTf). Fe(III)-bound transferrin docks onto the transferrin receptor 1 (TfR1), forming the TfR1-Tf-(Fe^3+^)_2_ complex, which undergoes endocytosis. The ferric reductase STEAP3 reduces Fe(III) to Fe(II), releasing Fe from sTf and enabling its transport out of the endosome through the divalent metal transporter (DMT-1). Fe enters into the cytosolic labile iron pool (LIP) and a certain amount is trafficked to ferritin (FTH), where it is stored, and the rest is trafficked to be incorporated into the biomolecular machinery of cells. Some cells, like intestinal cells, possess the membrane-bound transporter ferroportin 1 (FPN-1) used to export Fe out of cells to facilitate delivery to other cells and regulate the homeostatic level of the metal [15]. The human body lacks an Fe excretory process. A small but steady amount of Fe is lost through sweat, skin shedding through scratching, and shedding of intestinal cells. The menstrual cycle in women also results in Fe release. The lack of a regulatory excretion method makes Fe overload a very serious health concern. When the homeostasis of intracellular Fe is lost, an excessive buildup of Fe can result in an abundance of lipid peroxides, causing oxidative stress and ultimately leading to ferroptotic cell death (Figure 2, route 1) [15]. Furthermore, a sudden influx of labile Fe into cells, presumably through sTf-mediated endocytosis, can also induce ferroptosis [16].

Another well-known mechanism associated with ferroptosis involves amino acid metabolism (Figure 2, route 2). Cancer cells typically express the protein SLC7A11 (member 11 of solute carrier family 7) on the cell surface [15,17]. This protein regulates the Xc^−^ system, which acts as a cystine/glutamate transporter to exchange intracellular glutamate, reducing cystine to cysteine to synthesize glutathione (GSH). In turn, GSH is a necessary cofactor for glutathione peroxidase 4 (GPX4) to eliminate ROS. This prevents cells from experiencing excessive lipid peroxidation and cell death. Blockade of SLC7A11 results in the inhibition of GPX4 (Figure 2, route 2) [5,15].

Lipid metabolism (Figure 2, route 3) is the final common pathway in the initiation of ferroptosis. This mechanism involves the activation of arachidonic acid (AA), a fatty acid substrate, by long-chain acyl-CoA synthetase family member 4 (ACSL4), which dictates ferroptosis sensitivity. The activated AA produces AA-CoA, which is then esterified by lysophosphatidylcholine acyltransferase 3 (LPCAT3) to generate phosphatidyl-(PE)-AA. The process of lipoxygenases (LOX) oxidizing PE-AA results in the formation of cytotoxic PE-AA-OOH. The balance of GPX4 activity plays a crucial role in the regulation of lipid-OOH. An abundance of polyunsaturated fatty acid (PUFA) promotes the production of reactive oxygen species (ROS) and hazardous lipid peroxides while concurrently reducing GPX4 activity, initiating ferroptosis (Figure 2, route 3).

Regardless of the route of onset, ferroptosis features a characteristic biomarker profile: the GSH-GPX4-ROS-LPO axis. There is a decrease in GSH levels with a subsequent decrease in GPX4 activity and possibly a decrease in GPX4 expression. These behaviors are accompanied by an increase in ROS levels and an increase in lipid peroxidation (LPO) levels, malondialdehyde (MDA) levels, which increases the expression of the cytokine cyclooxygenase-2 (COX-2) and of γ-H2AX. Great insight into the manifestation of ferroptosis has been garnered through the discovery/development of ferroptosis inducers and inhibitors. The inducers consist of erastin (system Xc^−^ system inhibitor), the covalent GPX4 inhibitors (ML162 and (1S, 3R)-RSL3), mevalonate pathway (responsible for GPX4 maturation) inhibitors (FIN56 and analogues), promoter of iron oxidation ((−)-FINO_2_), and temozolomide (DMT-1 enhancer) [18,19]. The inhibitors include the iron chelators (deferoxamine, deferiprone, deferasirox), free radical-trapping antioxidants, GPX4 activators, and LPO inhibitors [19]. To date, this diverse set of chemical tools has yet to produce a clinical anticancer drug with the specific intention of inducing ferroptosis. Efforts to translate the ferroptosis inducers into potential anticancer agents have been limited by their lack of appropriate drug-like characteristics. The compounds tend to suffer from water solubility issues, and in vivo, they lack potency, selectivity, and metabolic stability [20,21].

Recent studies reveal that coordination chemistry and non-iron transition metals may unlock new avenues for the development of ferroptosis-inducing drugs, especially with regard to manipulating intracellular Fe activity and lipid regulation. While Fe is crucial to the initiation of ferroptosis, copper (Cu) and zinc (Zn), also essential metals, play significant roles in this process due to their correlative interaction with iron. In different biochemical contexts, the loss of the cellular homeostasis of Cu and Zn can offset Fe homeostasis and induce ferroptosis. This biochemical interaction shines light on how chelation may play a vital role in altering intracellular levels of Fe, Cu, and Zn and their functionality to switch on ferroptosis. It also more broadly reveals the potential for engineering coordination compounds of nonessential metals to exploit their distinctive chemical capacities and their corresponding ligands to trigger molecular mechanisms that lead to ferroptosis. This review will examine chelation strategies, metal nanoparticles, and small molecule coordination compounds that have ferroptotic properties to elucidate a novel ferroptosis-based anticancer drug design.

## 2. Iron and Cancer

The essential element iron is the most abundant transition metal in human beings, ranging between 4.2 and 6.1 g in adults [22]. It is largely found in biomolecules in the body serving a variety of vital functions, including oxygen transportation by the globin proteins, electron transfer and redox, and activation of oxygen and nitrogen [22]. Biologically, Fe exists in both +2 and +3 oxidation states. While the +3 state is most dominant in the open oxidizing atmosphere, within the human body, Fe fluctuates between the +2 and +3 states, regulated by key redox modulating small biomolecules and proteins. In the reducing environment of cells, the Fe(II) state is dominant.

While Fe is pivotal to human survival, it also plays crucial roles in cancer progression. Cancer cells are highly dependent on Fe for their division, repair, and metastasis [23–25]. In cancer cells, there is an overexpression of the transferrin receptor [26], which results in an increased uptake of Fe(III)-bound serum transferrin into cells and an increase in Fe in the labile iron pool (LIP) [27–29]. Fe-dependent enzymes are overexpressed, such as ribonucleotide reductase (RNR), which produces the building blocks for DNA synthesis and repair [24]. An overexpression of Fe-containing matrix metalloproteinases [25] contributes to the degradation of the extracellular matrix and, subsequently, the migration of tumor cells from the tissue/site of origin.

### 2.1. Iron Chelation and Cancer Therapy

**Figure 3.**
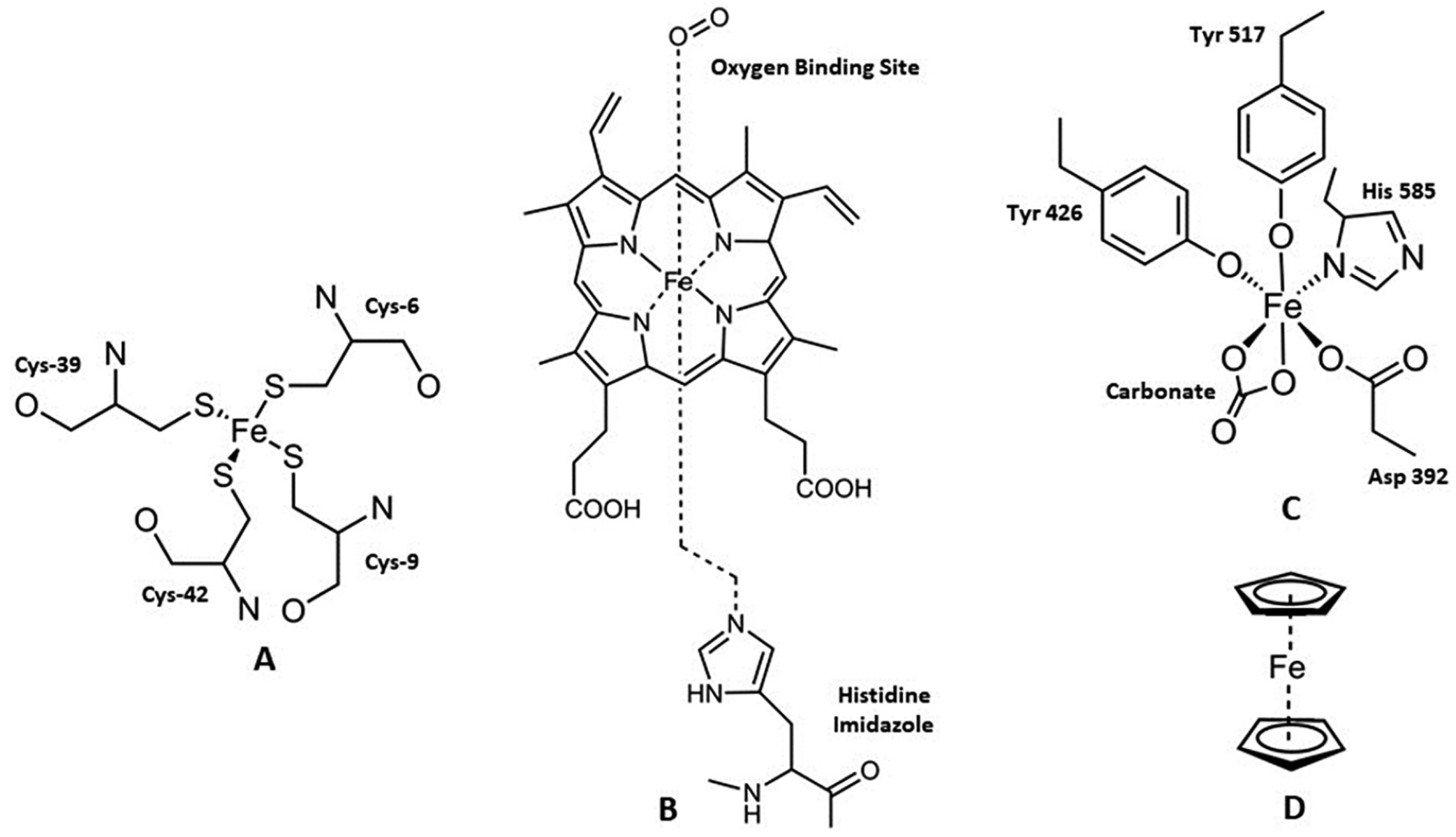
Representative iron coordination within rubredoxin (**A**), hemoglobin (**B**), human transferrin (**C**), and ferrocene (**D**).

The difference between the +2 and +3 oxidation states of Fe is such that the metal exhibits different coordination chemistry. In its +2 state, Fe is an intermediate Lewis acid with a d^6^ electron configuration [22]. Biological ligands tend to be S-atom- and N-atom-based, such as the proteins with Fe-S clusters and the heme-based proteins like hemoglobin and the cytochromes (Figure 3) [22,30]. Fe(II) typically forms octahedral complexes in high spin (S = 2), although there are examples of low spin (S = 0) complexes with ligands like 1,10-phenanthroline (phen), bipyridine (bipy), and cyano (CN^−^). Fe(II) also coordinates in other modalities, such as in Fe-S clusters, which feature tetrahedral coordination, and the classical ferrocene compounds, which can be described as a coordination number of 10 (Figure 3). In its +3 state, Fe is a hard Lewis acid with a d^5^ electron configuration [22]. Fe(III) complexes can vary between coordination number 3 and 8 but they are usually mononuclear octahedral compounds in high spin (S = 5/2) [22]. Like Fe(II), Fe(III) can form low spin (S = ½) compounds when coordinated to strong field ligand like phen, bipy, and cyano. Biological ligands of Fe(III) tend to be N-atom- and O-atom-based, as in the transferrin proteins, in which the metal binding site consists of the four amino acids: 2 tyrosines, 1 histidine, 1 aspartate, and bidentate carbonate, a synergistic anion (Figure 3). The ability to interconvert between the two oxidation states in certain biomolecules is biologically useful to tap into a variety of functions facilitated by the metal and the associated changes in the coordination modality.

**Figure 4.**
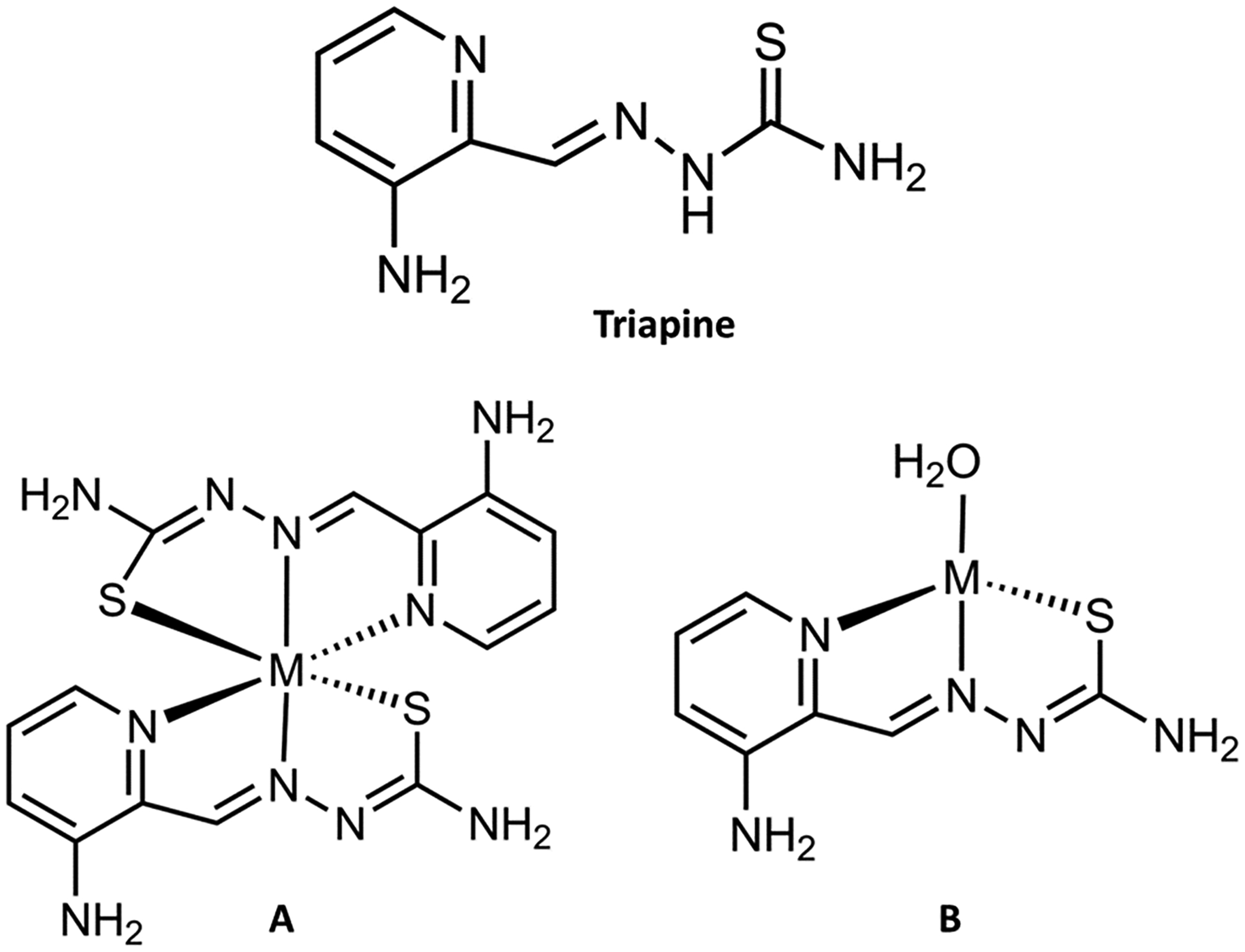
Triapine and a selection of its physiologically relevant metal compound species. At a near neutral pH of 7.4, Fe(II/III) forms the (**A**) modality, Cu(II) forms the (**B**) modality, and Zn(II) forms a mixture of the (**A**) and (**B**) modalities.

An important strategy to combat cancer has centered on the use of Fe chelators, which are molecular ligands that form multiple bonds with Fe by binding at two or more of their atoms. These chelators have very high affinity to the metal compared to other essential metals. Some Fe chelators work outside the cellular environment to block Fe(III)-transferrin cellular delivery, while others affect intracellular Fe and inhibit the metal’s bioavailability and innate functionality [31–33]. Some Fe chelators that have been tested for potential anticancer application are repurposed from their treatment of Fe-related diseases such as hemochromatosis [34], thalassemia, and sickle cell anemia [35]. These diseases can be either the result of excessive Fe buildup or the loss of Fe homeostatic control in an organ, and they can feature the induction of ferroptosis as a consequence of the Fe toxicity. Chelators used in these instances tend to feature N- and O-coordinating atoms (deferasirox, deferiprone, deferoxamine), which strongly bind Fe(III) and prevent its redox activity and thus its capacity to promote ferroptosis [36–38]. These chelators may also prove useful for attenuating the severity of neurodegenerative and heart diseases in which ferroptosis toxicity is observed [39–41]. The Fe chelator triapine, featuring NNS coordination (Figure 4), was specifically designed as an anticancer agent and its mechanism for handling intracellular Fe is distinct from the N- and O-based chelators.

### 2.2. Fe Chelation by Triapine Induces Ferroptosis

Since 1992, triapine (3-AP) has been the subject of over 30 clinical trials, where it has been investigated for its anticancer properties as an iron-binding agent [42]. It is a tridentate chelator that binds Fe(II) and Fe(III) ions with high affinity by means of two nitrogen donor atoms and sulfur, utilizing a thiosemicarbazone (TSC) scaffold. The log β of Fe(II)(3-AP)_2_ is 22.55 and the log β of Fe(III)(3-AP)_2_ is 26.3 [43]. The 1:2 metal:ligand species for both Fe(II) and Fe(III) dominates at the physiological pH of 7.4 (Figure 4). Moreover, 3-AP has demonstrated its potential as a highly effective cancer growth inhibitor, acting through a variety of pathways [42]. Primarily, it binds to intracellular Fe and forms the Fe(II)(3-AP)_2_ complex, which is responsible for inhibiting the enzyme ribonucleotide reductase (RNR) by reducing the critical tyrosyl radical located near a diiron cofactor site in the R2 unit of the enzyme [44]. A recent study demonstrated that Fe(III) complexes of 3-AP analogues, which exhibit a reversible redox couple less than E_1/2_ = 0.2 V vs. NHE and not greater than E_1/2_ = 0.5 V vs. NHE, could reduce the tyrosyl radical of RNR R2 [45]. Through this molecular mechanism, triapine induces cell death by way of apoptosis.

It was recently discovered that triapine can also induce ferroptosis. Zhang et al. explored a drug design strategy that involved the use of nanomicelles to co-deliver triapine and photosensitive chlorin e6 (Ce_6_) in hepatocellular carcinoma (HCC) [13]. The drug delivery system (DDS) was composed of lactose-coated nanomicelles (made from FlupDMAEMA-pAHPMA modified with fluorescein) loaded with the two anticancer agents, referred to as TCLM. For this study, they selected triapine because they hypothesized that it could trigger ferroptosis by generating excess hydroxyl radicals (•OH) via Fenton-like reactivity, which would lead to lipid peroxidation. To synergize with this behavior, they selected Ce_6_ to generate singlet oxygen (^1^O_2_) via near-IR (NIR) radiation. Using transmission electron microscopy (TEM) and dynamic light scattering (DLS), TCLM was found to be spherical in shape, with a diameter of 109.5 ± 6.4 nm. Fluorescence measurements were utilized to determine the drug loading capacity (DLC) and drug encapsulation efficiency (DEE). They calculated DLCs for triapine and Ce_6_ of 24.7 ± 1.8% and 22.2 ± 1.2%, respectively, using a LMs:Triapine:Ce_6_ feed ratio of 1:2:2. Meanwhile, the DEE for triapine and Ce_6_ was measured as 71.6 ± 3.1% and 52.2 ± 2.0%, respectively. They analyzed the release of 3-AP and Ce_6_ at different pH values. They observed that at a pH between 7.4 and 6.5, only 20% of the two were released in 24 h, while a more acidic pH between 5 and 5.6 released almost 80% [13], indicating a pH-dependent release mechanism. Electron paramagnetic resonance was used with TEMP as a capture agent to measure the generation of ^1^O_2_, which was only observed under NIR. As the pH decreased, the ^1^O_2_ production increased. The cytotoxic potency of TCLM was compared with that of 3-AP alone and 3-AP encapsulated in the nanomicelles (TLM) against non-cancer cells (HEK293 and L02) and HCC cancer cells (HepG2 and Huh7) [13]. Triapine demonstrated inhibition of cell viability against both the non-cancer and cancer cells. In comparison, the nanomicelles that included triapine had cancer-selective cytotoxicity. The nanomicelles that included both triapine and Ce_6_ exhibited the highest potency upon NIR activation. A series of parameters were analyzed to gauge whether TCLM was able to trigger ferroptosis. Using Western blot, they observed a decreased in GPX4 expression and an increase in COX-2 and γ-H2AX expression. They also measured an increase in lipid peroxidation (LPO) and a decrease in the glutathione levels [13]. Based on these promising results, they examined the effect of TCLM in BALB/c mice with xenograft HCC tumors. PDT was applied to the mice 7 days post-IV tail injection. It was observed that TCLM prevented growth of the tumor without affecting the mass of the animals or demonstrating any signs of toxicity. This study demonstrates the potential of a drug carrier in helping to shuttle more than one cell death agent specifically into cancer cells.

## 3. Cu and Zn Contribute to Regulating Fe Homeostasis

Both Cu and Zn contribute to the regulation of Fe in the body. The careful maintenance and biodistribution of Cu and Zn are crucial to Fe homeostasis [46]. Disruption of the Cu and Zn levels in the body can lead to Fe-related illnesses [47].

Cu exists in the body in both the Cu(I) and Cu(II) ion forms. Cu(I) is a d^10^ soft Lewis metal, whereas Cu(II) is a d^9^ intermediate Lewis metal. This difference in Lewis acidity is sufficient to dictate different coordination metal structures [30]. Cu(I) is commonly found in the trigonal planar (coordination number (C.N.) = 3) and tetrahedral modality, typically bound to S and N atoms. Cu(II) can form complexes of coordination number 4 (tetrahedral or square planar), 5 (trigonal bipyramidal or square pyramidal), and 6 (tetragonally distorted octahedral) bound to S, N, and O atoms. The capability of Cu to cycle between both oxidation states is an important property that underlies its redox activity and redox regulatory property as a co-factor of certain enzymes. One well-known family of enzymes is the multicopper ferroxidases (MCFs), which exhibit the significant role of Fe(II) conversion to Fe(III). The MCF hephaestin enables the exit of Fe from intestinal enterocytes via FPN-1 into the blood circulation in the +3 oxidation state to protect the body from unregulated Fenton-type reactivity of Fe(II) in the circulatory system [48].

**Figure 5.**
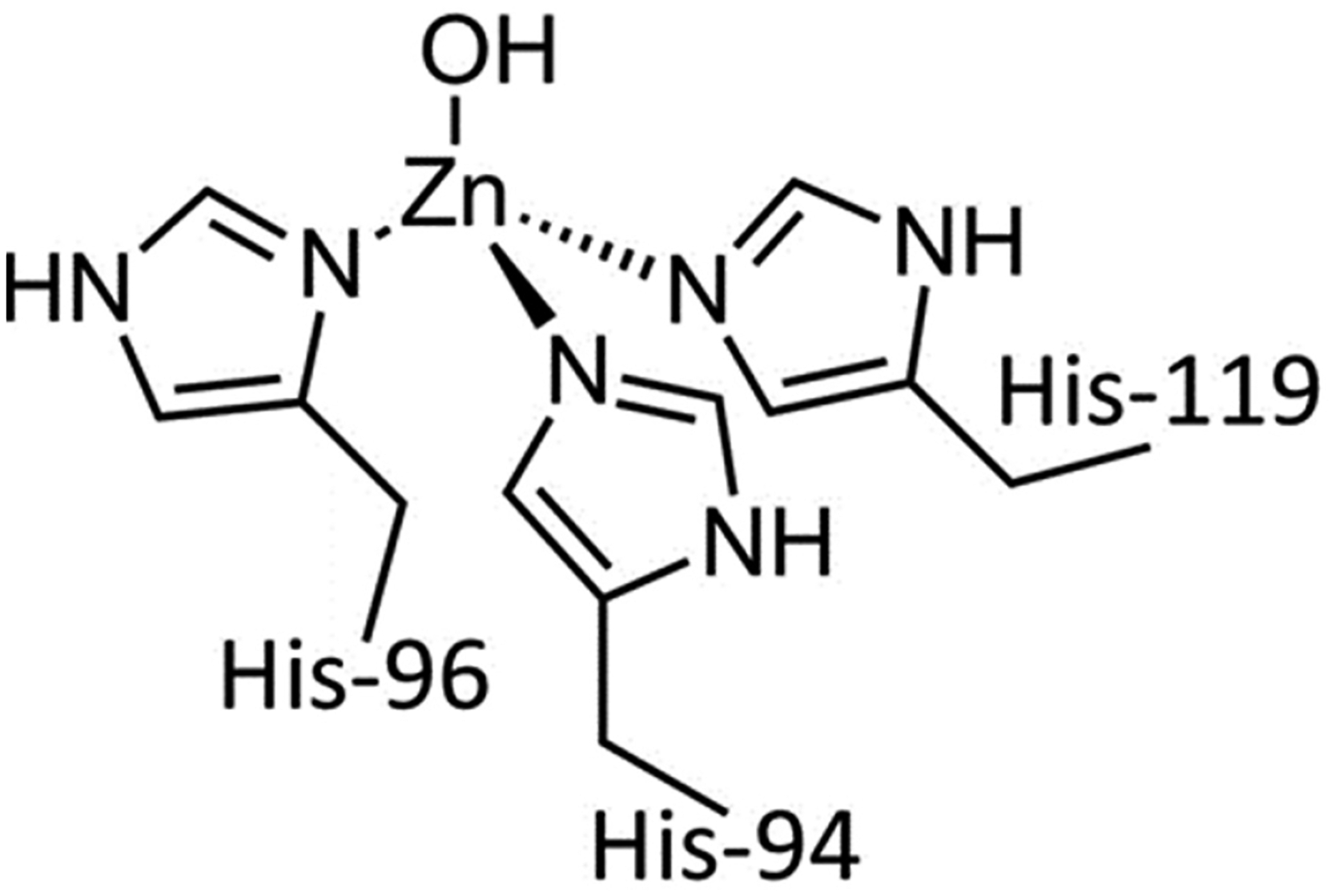
The Zn(II) binding site within human carbonic anhydrase II, demonstrating a tetrahedral coordination.

Among the transition metals, Zn is the second most plentiful element, and it typically adopts a sole steady oxidation state, Zn(II) (d^10^), which enables it to directly take part in a great variety of biological activities [22]. The coordination number for Zn(II) is typically four, arranged in a tetrahedral geometry as in its coordination in human carbonic anhydrase II, where it plays an acid-base regulatory function (Figure 5). Zn(II) is believed to play a part in Fe circulation. Given its redox inert status, Zn(II) is unable to regulate the oxidation state of Fe, unlike Cu(I/II). However, Zn(II) may modulate the expression of FPN-1 and DMT-1. Decreased pancreatic Zn(II) excretion reduces FPN-1 and DMT-1 expression and thus can affect Fe absorption and mobilization from tissues [49].

### 3.1. The Cu and Fe Interplay

**Figure 6.**
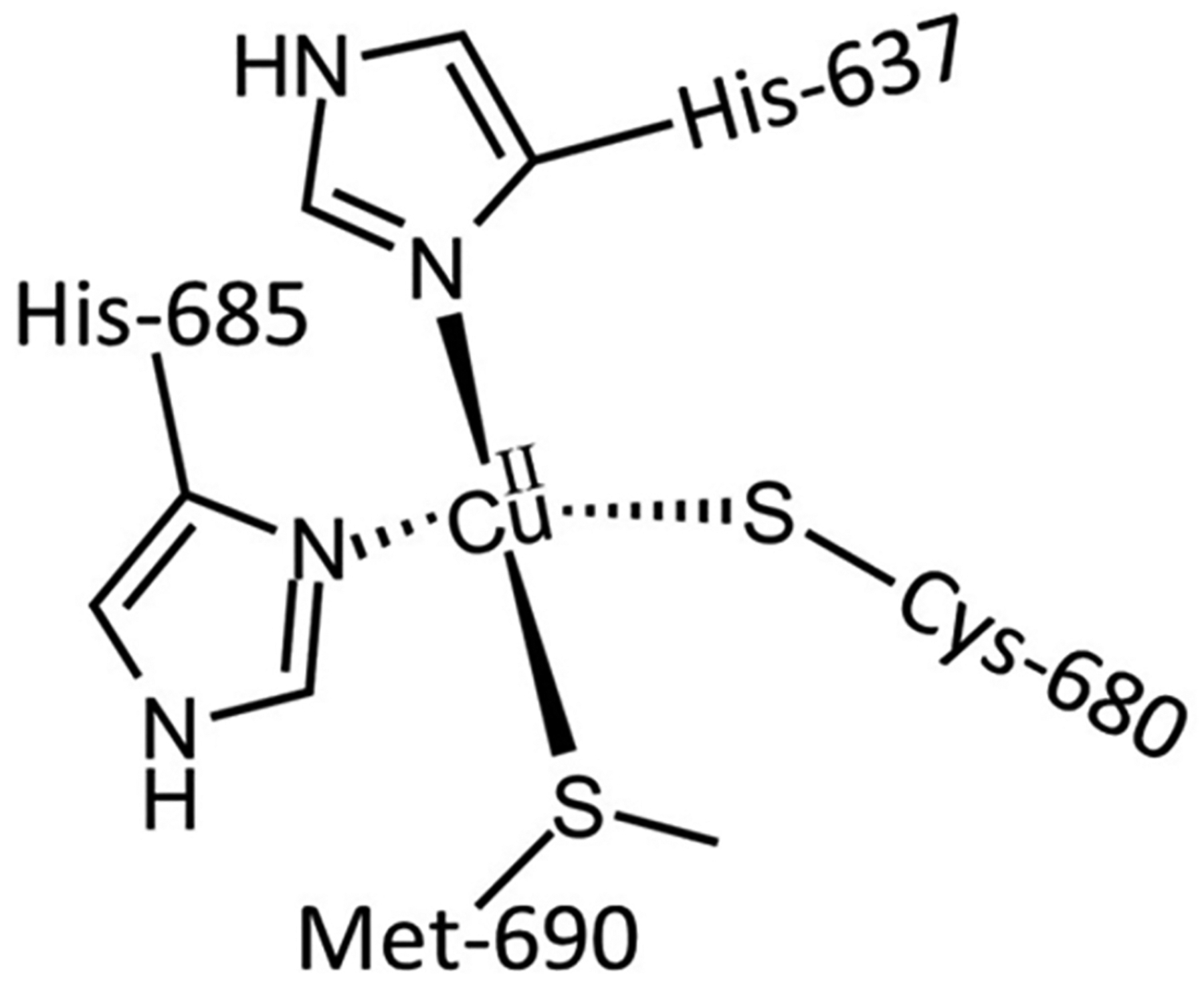
Human ceruloplasmin type 1 copper coordination. It is important to note that ceruloplasmin has six Cu binding sites, of which three are type 1 [30].

Cu ingestion and release from the body is carefully controlled by different molecular handling mechanisms, which make the metal not subject to physiological accumulation like Fe. Maintaining balanced levels of Cu in the body necessitates proper nutrition, absorption, distribution, and utilization, as well as excretion of any excess copper through bile. In the body, Cu is dominantly found in a protein-bound form and plays a major role in electron transfer as a co-factor in metalloenzymes [50]. In blood, about 95% of Cu is found in the MCF enzyme ceruloplasmin (CP) (Figure 6). The ATCUN site of human serum albumin accounts for the second most abundant amount of blood Cu (<5%). These proteins help to deliver Cu to cells via the copper transporter 1 (CTR1) in its +1 oxidation state.

In cells, Cu binds to several chaperone proteins that deliver the metal to specific sites to enable particular functions. Human CCS supplies Cu to Cu/Zn superoxide dismutase 1 (SOD 1) in the cytoplasm, which protects the cells from superoxide radicals [51]. Cyclooxy-genase 17 (Cox17) delivers Cu(I) into mitochondria, where it is inserted into the integral membrane enzyme cytochrome c oxidase (CcO). Antioxidant-1 (Atox1) traffics Cu(I) to the ATPase Cu transporters ATP7A and ATP7B. ATP7A transfers Cu from the portal blood into the liver’s circulation to deliver the metal to the liver, the primary storage location for Cu. ATP7B plays a crucial role in the liver by moving Cu from secretory vesicles into the bile to prevent Cu buildup [51,52]. Inherited disorders related to Cu metabolism, specifically Wilson disease (WD) and Menkes disease (MD), are caused by mutations in ATP7B and ATP7A, respectively. These disorders are known to result in severe neuropathological deficits, emphasizing the significance of Cu homeostasis in their manifestation.

Cu and Fe have an interrelatedness that is closely linked to the presence of MCF enzymes. CP, a Cu transporter, functions as a ferroxidase in serum. The liberation of Fe from storage in ferritin in response to a drop in the optimal level of Fe in the body requires the presence of CP for release into circulation. Studies have shown that a decrease in Cu levels has a direct correlation with a reduction in holoceruloplasmin production and an alteration in ferroxidase activity. When there is a decrease in the release of Fe in the tissues, it results in anemia. The only way to reverse this condition is by supplementing Cu, not Fe [53]. Dietary Fe absorption requires the proper functioning of hephaestin. The protein acts in conjunction with the IREG1 transporter to release Fe(III) from intestinal enterocytes for loading into transferrin [54].

### 3.2. Cu(II) Chelation/Complexation Can Induce Ferroptosis

**Figure 7.**
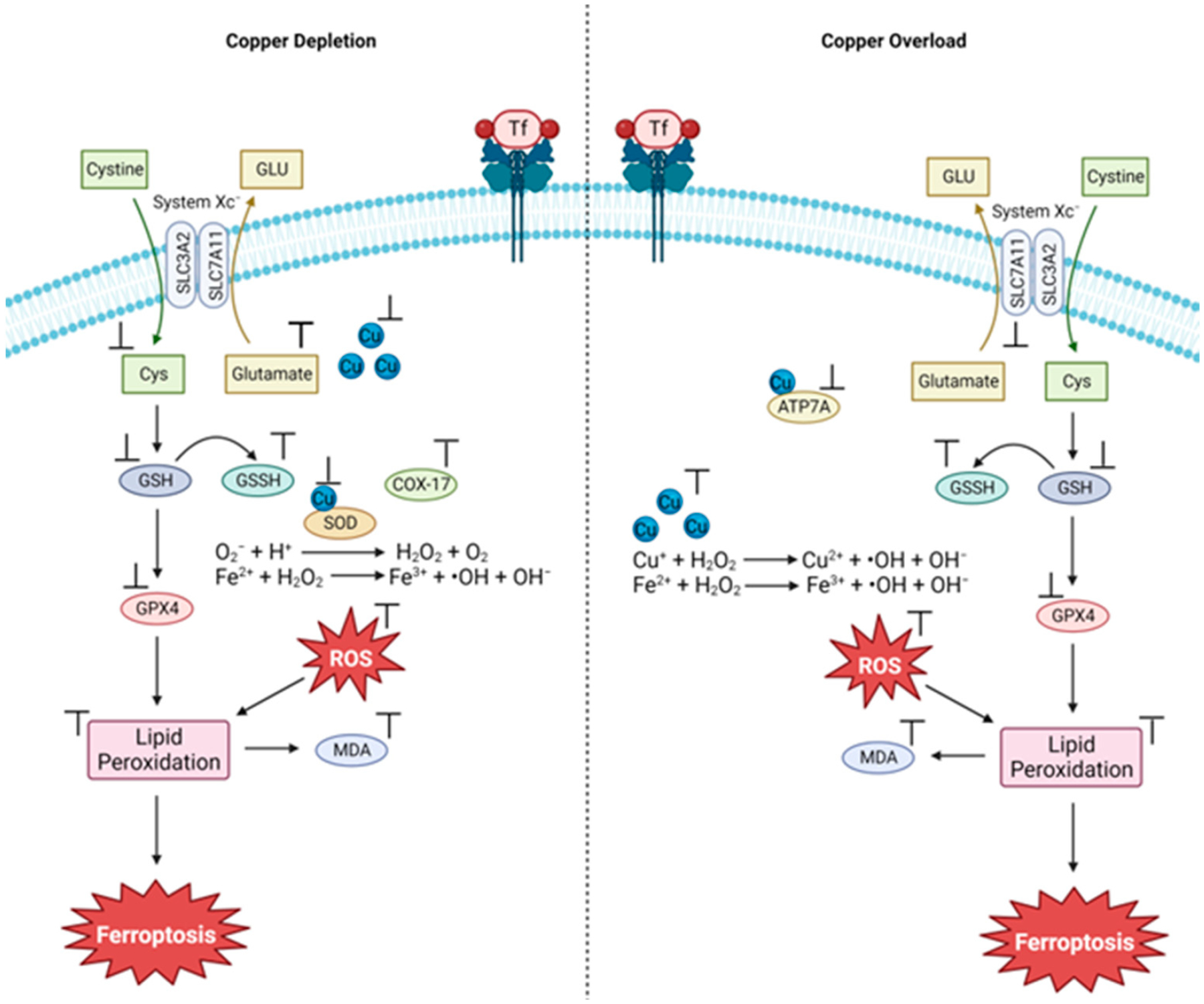
Proposed mechanisms of ferroptosis induction through copper depletion and overload (image created using Biorender.com).

Strategies that can control Cu cellular bioavailability may prove useful in treating cancer through ferroptosis (Figure 7). Cu depletion leads to a significant reduction in the activity of SOD1, the main antioxidant defense system of mammals, which can cause excessive accumulation of ROS. MCF deficiency can lead to changes in the iron balance in the body. This imbalance can lead to toxicity and lead to oxidative damage due to the accumulation of iron [48]. Excess Cu, in turn, inhibits the glutathione/oxidized glutathione ratio (GSH/GSSH) and GPX4, leading to excessive production of malondialdehyde (MDA), ROS and free Fe(II)ions, resulting in lipid peroxidation and increased membrane damage [50]. The increase in Cu(II) can also cause a direct degradation of GPX4, generating an accumulation of lipid peroxides, leading to ferroptosis (Figure 7) [55].

**Figure 8.**
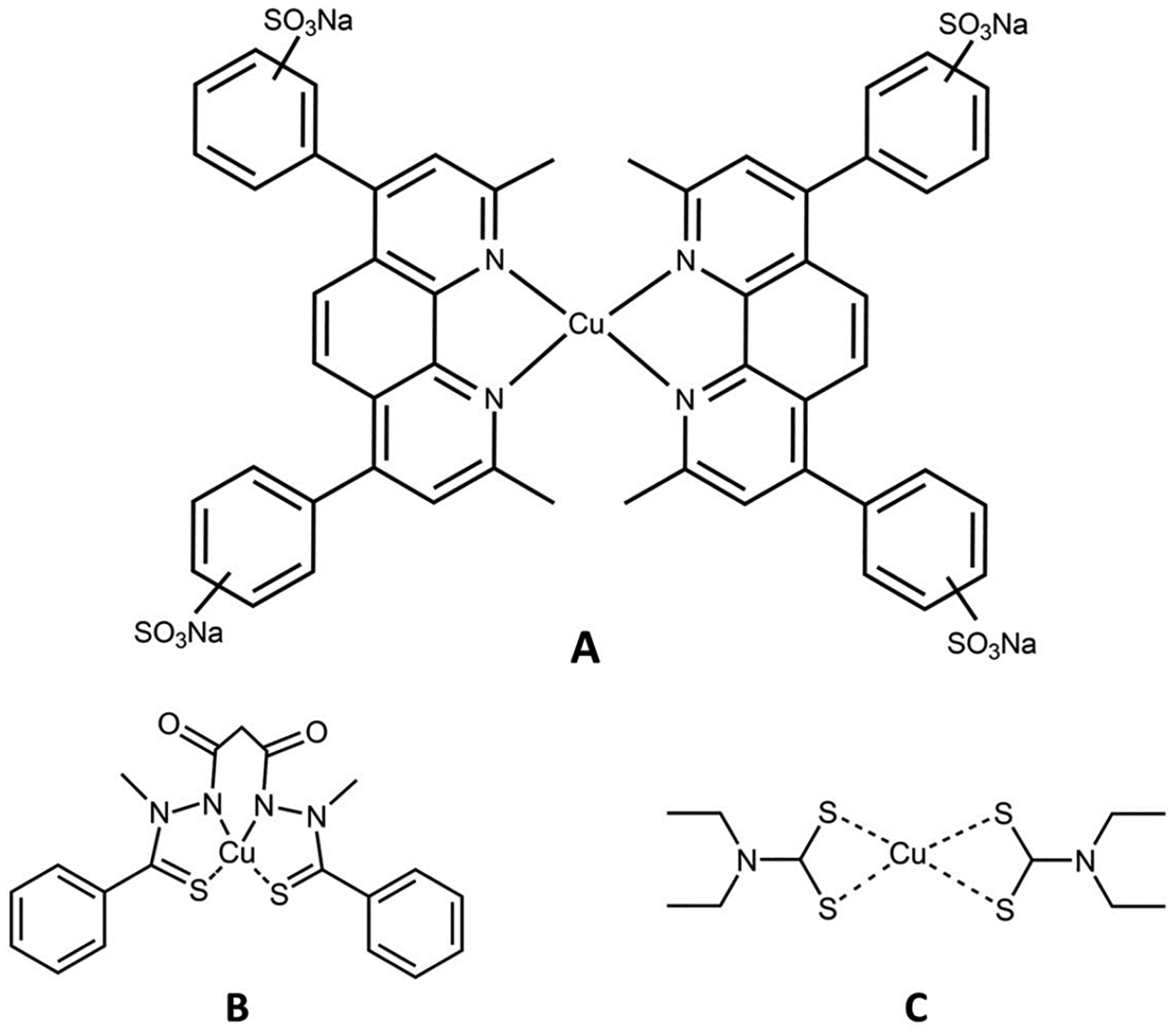
Cu(II) complexes of bathocuproynedisulfonic acid (**A**), elesclomol (**B**), and diethyldithiocarbamate (**C**).

Li et al. studied the effect of Cu depletion during ferroptosis. For this study, dermal papilla cells (DPC) were treated with the Cu chelator bathocuproynedisulfonic acid (BCS) (Figure 8A) [50]. A dose-dependent study was performed to determine the lowest amount of BCS that could deplete the intracellular Cu levels, which was determined to be 1000 μM after 72 h. At this concentration, there was a significant decrease in the level of CcO because of rapid degradation from depleted Cu and a subsequent increase in ROS species due to the depolarization of the mitochondrial membrane potential. A significant decrease in the activity of SOD1 resulted in an increase in superoxide radicals [50]. There was also a notable rise in the levels of LPO and MDA. Conversely, there was a significant decrease in the ratio of GSH to GSSH, and also a decrease in GPX4 activity [50]. When the ferroptosis inducer erastin was utilized, the observed cellular changes became even more pronounced. Interestingly, there was no significant increase in the intracellular Fe content, indicating that cell death was caused by Cu depletion, which enabled ferroptosis through the reduction of antioxidant capacities. This was confirmed through the use of the Fer-1 inhibitor, which decreased all the ferroptosis indicators [50].

On the other end of the spectrum, Huang et al. studied Cu(II)-overloading induced by elesclomol (ELC) in colorectal cancer (CRC) [14] and the possible connection with CRC inhibition. As a chemotherapeutic drug, ELC binds Cu(II) extracellularly (Figure 8B) and delivers the metal ion to mitochondria, where it undergoes reduction to Cu(I) and subsequently produces ROS [14]. During a 24 h period, the human colon adenocarcinoma cell lines SW480 and DLD-1 received treatment with either 2 μM CuCl_2_ or 20 nM ELC, separately or in combination. After this treatment, it was observed that cell viability was decreased by 40% with only ELC, but when combined with a Cu(II) salt, the viability was reduced by 60% [14]. The ROS levels increased 2-fold in DLD-1 cells and 3-fold in SW480 cells. The ELC and Cu(II) combined treatment depleted large amounts of reduced glutathione (GSH), especially within SW480 [14]. ELC also induced a decrease in GPX4 activity [14]. Upon treatment with various cell death inhibitors, ferrostatin-1 was the only one to significantly restore cell viability, indicative of ferroptosis induction.

Given that Cu(II) transportation is dependent on the ATP7A and ATP7B transporters located on the cytoplasmic membrane and mediated by Cu(II) efflux, the effect of ELC on the levels of these proteins was examined. ELC suppressed the level of ATP7A in CRC cell lines but not of ATP7B [14]. In vivo studies were performed in female nude mice with DLD-1 (colorectal adenocarcinoma cells) xenografts. Seven days postinjection, some of the mice received ELC (80 mgkg^−1^day^−1^) for 12 days. The mice that received ELC had, on average, half the tumor volume and weight than the vehicle-treated mice. The ELC appeared to serve as an antiproliferative agent, as it suppressed tumor growth. Immunohistochemistry (IHC) staining in the xenografts showed a decrease of about 35% in ATP7A expression in the ELC-treated group versus vehicle treatment, indicating that ELC downregulates ATP7A both in vitro and in vivo. IHC staining also revealed a downregulated protein level of SLC7A11 (transporter responsible for helping produce GSH) by a factor ~2.5 in the ELC-treated mice, characteristic of ferroptosis.

Another Cu(II) chelator pairing examined was that of a Cu(II) disulfiram complex (Figure 8C), Cu(II) diethyldithiocarbamate Cu(DDC)_2_ [56]. This complex is considered a potent anticancer agent due to its ability to inhibit NF-κB and the ubiquitin–proteasome system and alter intracellular ROS levels [56]. Du et al. investigated the mechanism by which Cu(DDC)_2_ causes death in hepatocellular carcinoma (HCC) [57]. Human HCC cells Huh7 and SMMC-7721, and normal hepatocytes L02, were treated with different concentrations of Cu(DDC)_2_ (0–3.0 μM) for 12 and 24 h. At 3.0 μM, a 60% decrease in viability was observed after 12 h and a complete reduction of cancer cells after 24 h. In the noncancer cells, there was only a 50% reduction after 24 h [57]. Cell death inhibitors were screened and the higher cell viabilities of cells treated with Cu(DDC)_2_ were observed in the presence of ferrostatin-1 and the iron chelator DFO, indicating that the primary cause of death was ferroptosis [57].

Normally, the mitochondrial network in Huh7 and SMMC-7721 cells is elongated. When exposed to elevated concentrations of Cu(DDC)_2_, the mitochondria of HCC cells fragmented and accumulated around the nucleus, thereby impairing the mitochondrial oxidative phosphorylation process. However, the phosphorylation levels of several components of the MAPK signaling pathway, p38, JNK, and ERK, were not affected and thus were not related to the observed cell death. Significant increases in the γ-H2AX, p53, and p21 levels were observed, indicating the occurrence of DNA double-strand breaks. This means that mitochondrial homeostasis was altered and oxidative stress occurred [57]. Cu(DDC)_2_ treatment extended the half-life of NRF2 and inhibited its degradation. Overexpression of Keap1 was observed to sensitize Cu(DDC)_2_-induced ferroptosis with an increase in ferroptotic events, including accelerated superoxide production and lipid peroxidation [57].

### 3.3. The Zn and Fe Interplay

In contrast to other divalent metals and owing to its redox inertness, Zn(II) does not necessitate oxidation or reduction for transmembrane transportation [22]. The zinc transporter family is composed of two distinct groups. The first is the Zrt-/Irt-like proteins (ZIPs) family, which increases the concentration of cytosolic Zn by absorbing Zn(II) from the intestine and transporting it from the extracellular space to the cytoplasm [58]. The second group is the ZnT proteins, which decrease the concentration of cytosolic Zn(II) by functioning as intracellular Zn(II) exporters. Zinc transporter 1 (ZNT1) is a membrane protein that is situated in the basolateral membrane and is responsible for transferring Zn(II) to the bloodstream [58]. Zn(II) homeostasis in the body is regulated through the excretion of Zn via feces [49].

It has been approximated that 10% of the human proteome consists of proteins that bind with Zn, playing a crucial role in both catalysis and structure [59]. Zn also partakes in important functions in the metabolism of proteins, lipids, nucleic acids and gene transcription [60]. It also serves as an intracellular secondary messenger [61]. Zn influences the modulation of intestinal iron absorption and the mobilization of tissue. Various studies using intestinal cell culture models have shown that Zn(II) has the ability to stimulate the absorption and transcellular transport of iron by promoting the expression of DMT-1 and FPN-1 [49]. During Zn deficiency, Fe accumulates within cells while hemoglobin concentrations are stunted [49]. Thus, the Zn levels in plasma are typically reduced in iron deficiency anemia [62]. Zn deficiency can lead to various adverse effects, such as a weakened immune system, delayed growth, poor wound healing, hair loss, diarrhea and delayed sexual maturity [63]. Zn(II) poisoning can also lead to a variety of health problems. Elevated Zn levels can cause Fe transmetalation in respiratory chain proteins, causing increased oxidative stress. This, in turn, generates large amounts of free Fe, which can lead to ferroptosis toxicity. Zn poisoning is able to activate the mTOR complex 2 (mTORC2) or other kinases that inhibit the functioning of the Xc^−^ system through phosphorylation. Alternatively, Zn may directly inhibit the Xc^−^ system, glutamate biosynthesis, or GPX4 [10].

The solute carrier 30 (SLC30) family of transporters is responsible for regulating the movement of Zn from the cytosol to other parts of the cell. Of these transporters, it was discovered that ZIP7, a member of the SLC39 family, which is responsible for increasing the concentration of Zn in the cytosol, is a vital component in the occurrence of ferroptosis [64]. The overexpression or activation of ZIP7 serves as a reliable biomarker for detecting cancer cells that are receptive to ferroptosis-targeted treatments.

### 3.4. Modulating the Ability of Zn(II) to Induce Ferroptosis

In its salt form (ZnCl_2_), Zn(II) displays nonspecific but low potency cell toxicity. Skaar et al. studied the effect of Zn(II) ions on non-small cell lung cancer (NSCLC) cells [10]. A549 cells were treated with 250, 500, and 1000 μM Zn(II) in a time-dependent manner. Following 6 h of all the treatments, there was a change in the ratio of GSH to GSSH. For the 250 μM treatment, the ratio reverted back to the original level after 24 h. However, this was not observed for the 500 and 1000 μM treatments. The Zn(II) was observed to interfere with the mitochondrial regulatory mTORC2/RICTOR signaling pathway, resulting in the phosphorylation of the Xc^−^ system. Collectively, these behaviors induced by Zn(II) resulted in ferroptosis, which could be curtailed by treatment with the iron chelator deferoxamine and antioxidant vitamin E [10].

**Figure 9.**
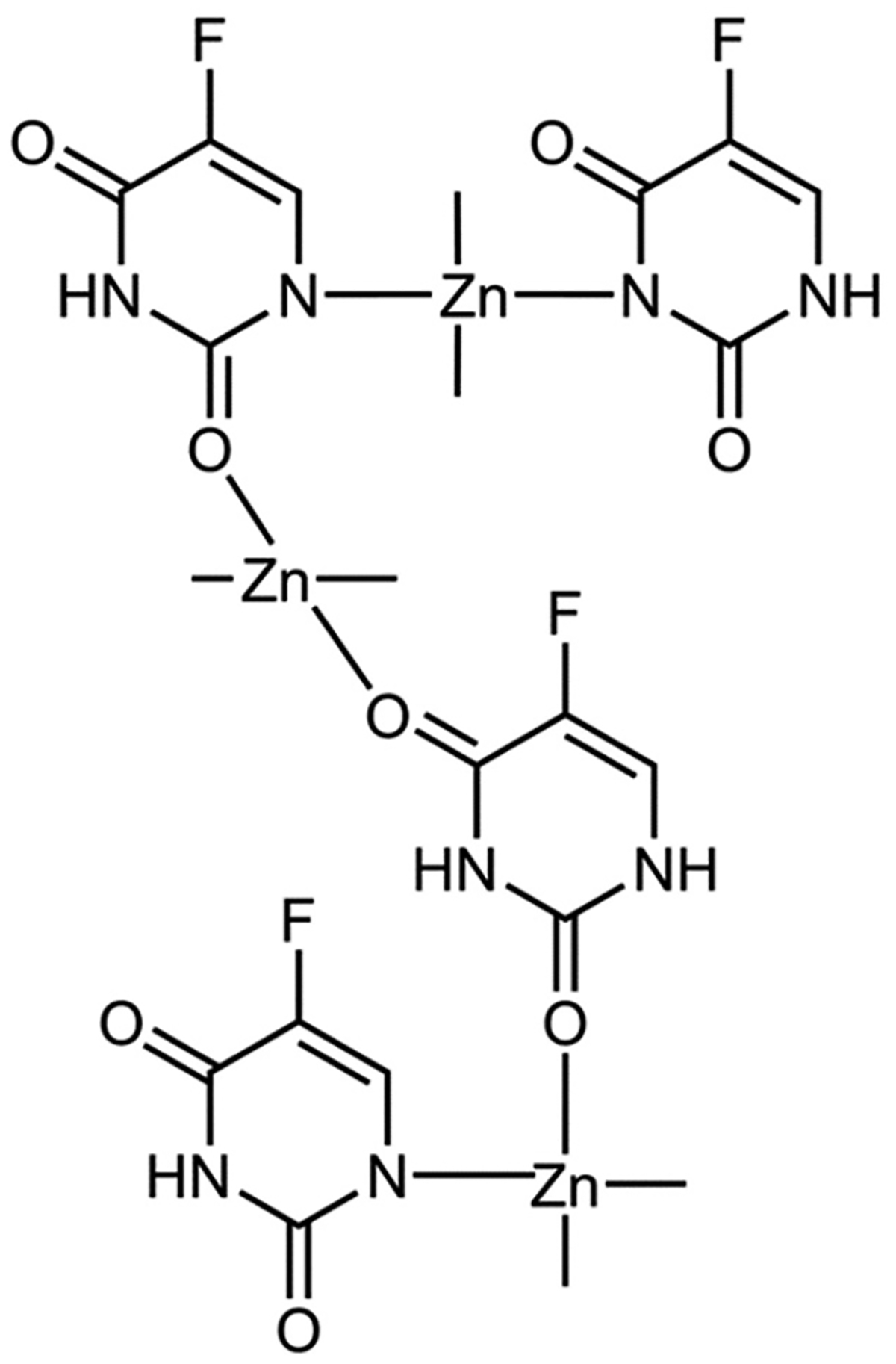
The Zn(II)-5-Fu metallodrug network (Zn-Fu MN) [65].

Zhang et al. explored the possibility of synergizing Zn(II) with the clinical anticancer drug fluorouracil (5-Fu) [65]. More specifically, 5-Fu is an antimetabolite chemodrug that mimics the building blocks of RNA and DNA and thus can disrupt the ability of cells to produce DNA and proteins. It also displays the ability to induce immunogenic cell death (ICD). In vivo, however, the drug exhibits a poor immune response, likely due to low selectivity. Nanoparticle vehicles have been designed to enhance 5-Fu cellular delivery and its immune response, but efforts have less than ideal. Due to the Zn(II)-binding capabilities of 5-Fu, Zhang et al. rationalized that a Zn(II) 5-Fu polymeric-like network could be developed that would combine the cell intrusive properties of both Zn(II) and 5-Fu and also facilitate the ^19^F-magnetic resonance imaging (MRI) “turn-on” potential due to the high drug payload within cancer cells [65]. A Zn(II)-5-Fu metallodrug network (Zn-Fu MNs; Figure 9) was created through a straightforward process of mixing Zn(NO_3_)_2_ and fluorouracil by means of sonication. Through the use of transmission electron microscopy (TEM) and dynamic light scattering (DLS), it was demonstrated that the Zn-Fu MNs had a slender and threadlike structure, with a diameter size of ~100 nm and zeta potential of −6.96 mV, indicative of a negative surface charge. Thermogravimetric analysis (TGA) measurement revealed that the mass percentage of 5-Fu in the Zn-Fu MNs was 40.93%, highlighting the potential for substantial drug-loading capabilities [65]. Drug release was examined under tumor microenvironment (TME) conditions. The combination of a low pH (pH 5.4) and a high ATP concentration (5 mM) resulted in virtually 100% dissociation of Zn(II) and 5-Fu from a 0.4 mg/mL amount in less than 30 min and shrank the diameter of particles to 5 nm. This dissociation was correlated with turning on of ^19^F-MRI [65].

The Zn-Fu MNs were analyzed in vitro in a murine colorectal carcinoma cell line (CT26) [65]. Zn-Fu MN (50 μg/mL, containing 20 μg/mL 5-Fu and 10 μg/mL Zn(II)) was added to the CT26 cells [65]. Using DCFH-DA as a probe, an increase in the ROS levels was observed in the Zn-Fu MN-treated cancer cells compared to treatment with PBS buffer control and 5-Fu alone. The ROS level increase exceeded that of the 10 μg/mL Zn(II) alone treatment. After treatment with the complex, the intracellular GSH concentration in the cancer cells decreased by 25%, more so than observed for Zn(II) alone. When the concentration of Zn-Fu MN was raised from 50 to 150 μg/mL, it was observed via Western blot that the signal intensity of the GPX4 protein gradually diminished, indicating the impact of the concentration on the protein [65]. At 50 μg/mL Zn-Fu MN, an approximate 52.95% escalation in the lipid peroxide levels was observed compared to 48.48% for Zn(II) alone as well as an amplification in the formation of MDA and γ-H2AX species. All these results indicate ferroptosis-induced cell death [65].

In order to achieve effective anti-tumor immunity, it is important to augment the initial activation of T cells and their infiltration into the tumor. After administering Zn-Fu MN to CT26 cells, there was a significant increase in the infiltration of CD8^+^ T cells and CD4^+^ helper T cells with cytotoxic properties in the tumors compared to the control groups [65]. This clearly demonstrates a heightened immune response. CT26-tumor-bearing BALB/c mice were utilized for the immunized treatment via subcutaneous injections of PBS, 5-Fu (100 μg/mouse, 20 μL), Zn(NO_3_)_2_ (50 μg/mouse, 20 μL), or Zn-Fu MN (250 μg/mouse, 20 μL, contains 50 μg Zn(II) and 100 μg of 5-Fu) on days 0, 2, and 4. The tumor growth curves were observed for 16 days, and it was discovered that Fu and Zn(II) were able to partially impede tumor growth in comparison to the PBS group. Remarkably, the tumors in the Zn-Fu MN groups were completely eradicated. Additionally, there was no significant weight loss among the mice in all the treatment groups during the treatment for cancer [65].

### 3.5. Considering the Cu(II) and Zn(II) Coordination of Triapine and Derivatives

Given the biochemical relationship of Fe with Cu and Zn, it is important to consider the Cu(II) and Zn(II) complexes of 3-AP and 3-AP analogues and their relevant anticancer potential. At a physiologically relevant pH 7.4, 3-AP type ligands form 1:1 metal:ligand Cu(II) and complexes in a distorted square planar geometry in which aqua/hydroxo ligands bind at the fourth site (Figure 4, form B) [42,66–68]. Zn(II) forms a mixture of the 1:1 metal:ligand and 1:2 metal:ligand species (Figure 4, forms A and B) [67]. The log β of the Cu(II)(3-AP) 1:1 species is 17.57 [68] and the Zn(II)(3-AP) 1:1 and 1:2 species is 8.78 and 16.26, respectively [67]. Moreover, 3-AP has very poor affinity for Cu(I) at pH 7.4, as indicated by the irreversible reduction of Cu(II)(3-AP) (E_c_ = −320 mV vs. NHE) [42].

Popović-Bijelić et al. compared the cytotoxic capability of triapine with that of its Fe(III), Ga(III) (an Fe(III) mimic), Cu(II), and Zn(II) complexes [69]. For this study, the human 41M (ovarian carcinoma) cell line was selected. Triapine exhibited an IC_50_ value of 0.45 μM, whereas the Zn:3-AP 1:1 complex and Ga:3-AP 1:2 complex exhibited respective values of 0.52 and 0.25 μM. These values of the metal–complexes seem to reflect a ligand-only activity, as they are virtually identical to metal free triapine based on the ligand concentration. These results indicate dissociation of the metal ions at the submicromolar concentrations and no effect of the free metal ions at these concentrations. The Cu:3-AP 1:1 and Fe:3-AP 1:2 complexes exhibited the same IC_50_ value of 1.5 μM, inferior to the free ligand by a respective factor of ~3 and ~6 when comparing to the concentration of the ligand. By already being Cu(II)- or Fe(III)-bound, the ligand can display redox activity but has a suppressed capacity to bind and diminish the intracellular LIP, which is dominantly Fe(II)-based.

Arion et al. prepared Fe(III) and Cu(II) complexes of the pyridinemidrazone and S-methylisothiosemicarbazone ligands analogues of 3-AP [66]. The speciation of the compounds were as expected: 1:1 Cu(II) ligand and 1:2 Fe(III) ligand. The cytotoxic properties of the compounds were examined in A2780 ovarian cancer cells and the cisplatin-resistant variation (A2780cis) and HEK293 non-cancerous cells. All the metal compounds were more potent than the free ligands but were virtually equally cytotoxic to the cancer and non-cancerous cells. The Fe(III) S-methylisothiosemicarbazone compound displayed the capacity to redox cycle and presumably would operate like Fe(II)(triapine)_2_ in terms of its mechanism of cell death. The Cu(II) complexes display irreversible reduction to Cu(I). In the presence of 12.0 mM GSH and 100 μM Cu(II) complexes, Cu(II) is quickly reduced to Cu(I), resulting in free ligands, as observed using UV-Vis spectroscopy [66]. It was observed via inductively coupled plasma mass spectrometry (ICP-MS) analysis that A2780 cells treated with 2.5 μM Cu(II) S-methylisothiosemicarbazone compound and 20 μM Cu(II) pyridinemidrazone compound resulted in the same amount of cellular Cu (~1 nmol Cu/mg of protein). This finding indicated that the improved cytotoxicity of the S-methylisothiosemicarbazone compound (IC_50_ of 1.5 μM; 10 times lower than the other compound) may be due to a more efficient intracellular delivery or less efficient cellular efflux of Cu(II) [66]. This influx of Cu(II) could potentially trigger ferroptosis via two possible mechanisms. It has previously been shown that Cu(II) can catalyze the oxidation of GSH, forming GSSG and resulting in the consumption of GSH [70], and can degrade GPX4 [55].

Extensive modifications of the triapine structural template have been achieved in which the chelators induce Cu-centered [71] and Zn-centered [72] cell death. While interesting, these chelators fall outside the scope of our study.

From these collective examples of chelators facilitating Cu- and Zn-centered influence on ferroptosis, it is obvious that the great benefit of the ligands lies in finetuning the bioactivity of the metals. That said, many of the chelators do not have a structural moiety that would facilitate specificity for targeting cancer cells and thus are likely to operate in a similar capacity within noncancer cells. In the next section, we explore how nanoparticles can help facilitate the cancer cell targeting of the Cu and Zn ferroptotic systems.

### 3.6. Ferroptotic Capacity of Zn and Cu Nanoparticles (NPs)

The Zn-Fu MN example spotlights the value of nano-sized systems for delivering a high metal payload into cells to trigger ferroptosis. In general, nanoparticles (NPs) are widely studied as anticancer drug delivery vehicles because of their enhanced permeation and retention effect (EPR) [73]. NPs offer the advantage of targeting tumors because the vasculature surrounding the tumor cells are very porous compared with healthy cells and particles of a diameter size between 10 nm and 400 nm can accumulate in the cytoplasm of tumor cells after cellular uptake. Diffusion back into the bloodstream is slow, resulting in a higher retention of the NPs within cells [74]. NPs made with ferroptotic metals would function in the dual role of drug and drug delivery agent. Several types of Fe-based NPs, some with surface functionalization for cell membrane receptor uptake, have been evaluated for their ferroptosis-inducing capability. They are believed to operate principally through pH-induced release of Fe into the cytosol, presumably via lysosomal degradation. For information on Fe-based NPs we refer you to review articles [75,76]. Zn- and Cu-based NPs are also being designed for ferroptosis, which takes advantage of the distinct chemistry of the metal centers.

**Figure 10.**
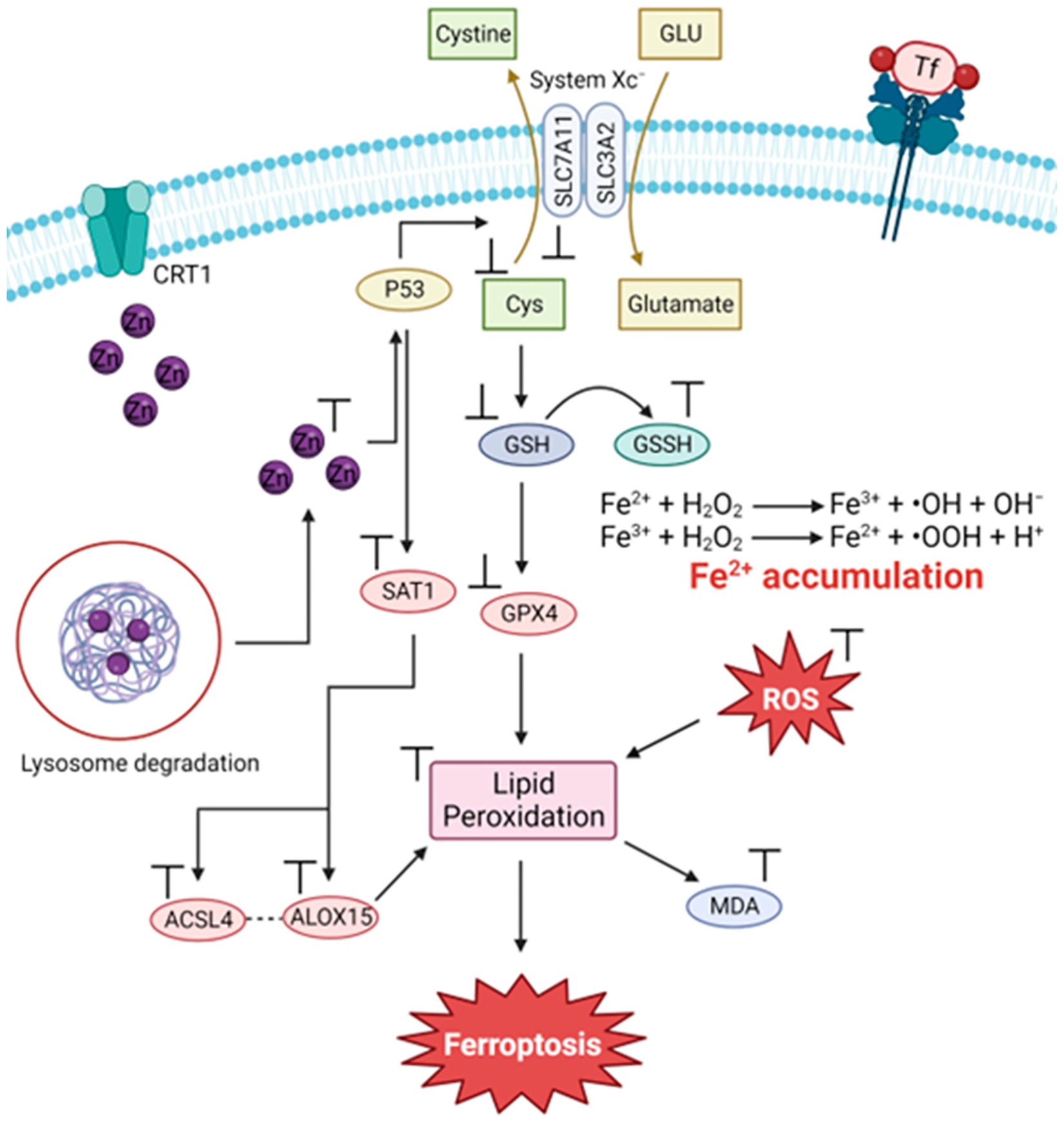
Proposed mechanisms of ZnO-NP’s induction of ferroptosis (image created using Biorender.com).

ZnO-NPs may be engineered to be used in various types of cancers. In a study examining the potential of Fe-free NPs to generate ferroptosis toxicity not necessarily specific to cancer, ZnO-NPs were assessed because of their use in nanomaterials for human cosmetic wear, nutritional intake, and biomedical applications [77]. Song et al. labeled commercially available ZnO-NPs with fluorescein isothiocyanate (FITC) for ease of tracking the NPs. The F-ZnO-NPs possessed the respective hydrodynamic size and zeta potential of 380 ± 5.6 nm and −18.6 ± 0.1 mV [77]. Upon entry into human umbilical vein endothelial (HUVEC) cells, a vast majority of the ZnO-NPs (~60%) underwent prompt degradation, due to the lysosome and transformed into Zn(II) ions, which, as noted previously have ferroptosis-inducing capability (Figure 10). The NPs produced a dose-dependent decrease in cell viability and perturbed the Fe homeostasis in the cells. The levels of TFR1, DMT-1, and FPN-1 were significantly increased [77]. A reduction in the GSH levels and an increase in the accumulation of ROS was observed [77]. This, in turn, led to a decrease in the GPX4 levels, causing the formation of lipid peroxides and MDA to increase. The voltage-gated anion channel (VDAC) protein significantly increased after ZnO-NP treatment. This changes the permeability of the outer mitochondrial membrane. Zn(II) overload-induced mitochondrial lesions can exacerbate the dysregulation of dynamic metal stores, resulting in elevated intracellular iron and, ultimately, ferroptosis [77]. While this particular study centered on the use of noncancer cells for assessing the general toxicity of ZnO-NPs, it provides insight into its potential for anticancer ferroptosis-based strategies.

Cervical cancer is one of the most prevalent forms of cancer and results in a significant number of deaths. It is thought to arise from stem cells [78]. Playing a crucial role in tumorigenesis, metastasis, and even the resistance to cancer therapies, CSCs are a subset of small cells. These cells have the ability to form tumor spheroids, which are utilized in measuring the percentage of CSCs that exist within the tumor population and in studying the cells’ regenerative capabilities [79]. Sheng Lei et al. studied the effect of ZnO-NPs on cervical cancer CSCs and the capacity to induce ferroptosis [80]. They focused on identifying the mechanistic link between the cluster of differentiation 164 (CD164) and ferroptotic cell death via nanoparticle treatment [80]. CD164 is a signaling receptor that regulates proliferation, adhesion and migration in hematopoietic stem and progenitor cells. It is also thought to be an oncogene [81]. ZnO-NPs were synthesized via refluxing the precursor zinc acetate dihydrate (0.1 M) in 1,2-ethanediol and triethylene glycol at 220 °C in the presence of sodium acetate (0.01 M) for 3 h. The ZnO-NPs had an average particle size of 38 nm, a zeta potential of −20 mV and conductivity of 0.1 mS/cm [80]. The ZnO-NPs were administered to Hela and C33A cervical cancer cells. The C33A and Hela cell viabilities were inhibited by ~50% after 4 days [80]. In both cell lines, a decrease in the GSH levels and in GPX4 expression and a concomitant increase in ROS and MDA were observed, in addition to an increase in the intracellular Fe levels. These behaviors were reversed through cellular treatment with Fer-1, indicative of ferroptosis cell death. It was observed that ZnO-NPs inhibits the expression of CD164 by promoting microRNA-506-3p tumor suppressor genes in cervical cancer cells. The addition of CD164 reversed ferroptosis, increased GSH, and decreased the iron levels as well as ROS and MDA lipids [80].

In vivo analysis of BALB/C mice was performed with three groups (control, ZnO-NPs, ZnO-NPs + OE CD164) [80]. C33A and HeLa cells were transplanted into the mice. The tumor size and body weight of the mice were measured every 5 days for 30 days. The ZnO-NPs inhibited cervical cancer cell growth by targeting CD164. The tumor weight in the mice treated with ZnO-NPs was ~2.5 times less than in the vehicle-treated mice. However, in the mice overexpressing CD164, the effects were reversed [80].

NPs based on Cu take advantage of the metal’s redox activity and ferroptosis-inducing capability [82]. A particularly interesting example is the liposomal Cu(ll) pectin NP (CuCP Lipo NP) [83], designed to operate in a multimodal localized fashion by incorporating components for photothermal therapy (PTT) and catalytic therapy. PTT converts absorbed light energy into heat energy to produce thermal burns locally at the tumor site. Although PTT is quite effective, it can cause severe cell necrosis from excessive heating and thus have a nonspecific effect within the body. To control for this potential detrimental effect, it is beneficial to couple this technique with a complementary/synergistic approach that can maintain the heating exposure time and intensity at lower levels. To this extent, catalytic therapy may prove useful. This strategy involves the generation of toxic free radicals mediated by enzymatic reactions in the TME. This has been shown to have an additive effect on PTT [84]. The emergence of peroxidase (POD) nanozymes has facilitated the catalyzation of H_2_O_2_ in the TME to create hydroxyl radicals (•OH), which exhibit high cytotoxicity. These radicals have the potential to induce varied death pathways within cancer cells.

Yuan et al. designed nanoenzymatic nanoparticles that contained Cu(II) [83]. Cu(II) 5,9,14,18,23,27,32,36-octabutoxy-2,3-naphthalocyanine was dissolved in THF and then DPPC, DSPE:PEG-2000-Mal, and cholesterol with an optimized weight ratio 3:1:1 were added in sequence. The solution was sonicated and then dropped into deionized water. CuCP Lipo NPs were formed after removing the THF by means of rotovap. Using TEM, the NPs were observed to exhibit homogeneous quasisphere structures. They have a diameter of 107 nm and a zeta potential of −27 mV [83]. The CuCP molecules were encapsulated with a maximum efficiency of approximately 80%. Additionally, the UV-Vis-NIR absorption spectra analysis of the NPs revealed a prominent absorption peak in the 800–900 nm range [83]. The NPs exhibit good photothermal capability by converting NIR irradiation into heat energy. They also catalyze H_2_O_2_ production of •OH via the Fenton reaction facilitated by the Cu(II) centers being reduced to Cu(I), consuming GSH to produce GSSG in cells. CT26 colon cancer cells and MKN45 gastric cells exhibited a decrease of 80% and 90%, respectively, in their GSH levels after being treated with 50.0 ug/mL CuCP Lipo NPs for 24 h and exposed to NIR irradiation (10 min, 0.8 W cm^−2^). Fluorescence imaging showed that the catalytic activity of CuCP Lipo NPs increases the ROS levels in both cell lines. When subjected to low concentrations in this study (6.12 to 12.5 ug/mL) of NPs plus NIR irradiation (808 nm, 0.8 W cm^−2^), the viability of MKN45 and CT26 cells showed a decrease of 60% compared to those not irradiated. However, at large concentrations (50.0 ug/mL), the NPs exhibit general toxicity without the need to use irradiation [83].

In addition to reducing the intracellular GSH levels, a reduction in the intracellular expression of GPX4 was observed in both cancer cells. Malondialdehyde (MDA) and 4-hydroxynonenal (4-HNE) were increased, reflecting increased intracellular lipid peroxidation levels [83]. The collective results provide conclusive evidence that the use of CuCP-Lipo-NPs can induce ferroptosis in tumors cells and that the addition of near-infrared radiation can enhance the effects of ferroptosis. Annexin V and propidium iodide staining detected apoptotic cells caused by the NIR irradiation and confirmed to be the cause of the ROS stress that led to apoptosis. Moreover, through proteomics analysis, the pathways of apoptosis, ferroptosis and ROS synthesis were identified [83]. The p53 upregulation as an indicator of apoptosis and ferroptosis, transferrin receptor protein 1 upregulation regarding ferroptosis, and downregulation of glutathione S-transferase (GST) proteins suggested ROS synthesis. These findings together validate the successful development of a CuCP Lipo NP that can demonstrate biocompatibility, catalytic activity, photothermal activity, in vitro cytotoxicity, antitumor activity, and the ability to synergistically trigger the ferroptotic and apoptotic pathways [83].

An in vivo study was conducted on Balb/c nude mice subcutaneously inoculated with CT26 cells. Cyanine 5 (Cy5) fluorescent labeled CuCP Lipo NPs were intravenously injected (tumor size of ~200 mm^3^) [83]. The mice were separated into four groups: blank, NIR laser only, CuCP Lipo NPs only, and CuCP Lipo NPs plus NIR irradiation. The mice from the NIR laser only and CuCP Lipo NPs plus NIR irradiation groups were exposed to an 808 nm laser 12 h postinjection. Irradiation was performed on day 2 and 4 and the tumor size was recorded each day. It was observed that the CuCP Lipo NP alone suppressed tumor growth by a factor of two compared to the vehicle-treated mice. The NIR only group had insignificant tumor shrinkage. However, the combination of CuCP Lipo NP with NIR resulted in total tumor shrinkage [83]. The NIR only group had insignificant tumor shrinkage.

## 4. Metal Complexes That Induce Ferroptosis for Anticancer Application

The induction of ferroptosis through small molecule metal complexes is an emerging anti-cancer strategy. Metallodrugs possess distinctive characteristics because of their metal and ligand composition. Different metal centers can showcase varying coordination geometries and redox capabilities, whereas ligands may also demonstrate diverse biological activities. Furthermore, biothiols have a strong attraction to metal ions, and this interaction can potentially disrupt the cellular redox balance and impair protein and enzyme function. Light exposure sensitizes certain metal complexes, leading to the creation of ROS and singlet O_2_. This is due to the spin–orbital coupling, which heightens the probability of intersystem crossing. As a result, metal complexes have the potential to disrupt the balance of redox reactions and build up lipid peroxidation, ultimately leading to the occurrence of ferroptosis. In this section, we will explore some classical drugs that have recently been discovered to induce ferroptosis and also survey a selection of the newer metal compounds with varying chemical capabilities that can trigger ferroptosis. We will highlight the particularities of the cellular conditions that enable ferroptosis to be possible by some of these compounds in certain cell lines and not in others.

### 4.1. Cisplatin and Oxaliplatin

Following the FDA approval of cis-diammine dichloroplatinum(II) (cisplatin) in 1978, platinum(II) compounds have been lead chemotherapy drugs. The second-generation drug carboplatin and third-generation drug oxaliplatin are approved worldwide and are used in up to 50% of cancer treatment regimens. The long-term use of Pt(II) drugs in cancer treatment in many patients is limited due to their lack of selectivity to cancer cells causing nephrotoxicity, myelotoxicity and ototoxicity. Between 40 and 80% of patients have experienced permanent hearing loss due to cisplatin. Pt(II) operates as an anticancer agent principally by forming guanine crosslinks with DNA and also monoadducts with guanine [85], resulting in DNA breaks that ultimately trigger apoptosis. Pt(II) also forms other biomolecular adducts in a non-selective manner in both cancer and noncancer cells, which is a major source of systemic toxicity. Another major limitation of cisplatin is the acquired cellular resistance of cancer cells. Multi-drug resistance proteins are overexpressed to efflux the metal. In this vein, glutathione binds to Pt(II) via its cysteine thiol group and removes the metal through the multidrug resistance protein MRP2. Furthermore, cells activate a number of repair mechanisms, such as the nucleotide excision repair (NER), which repairs DNA and inhibits apoptosis [85].

**Figure 11.**
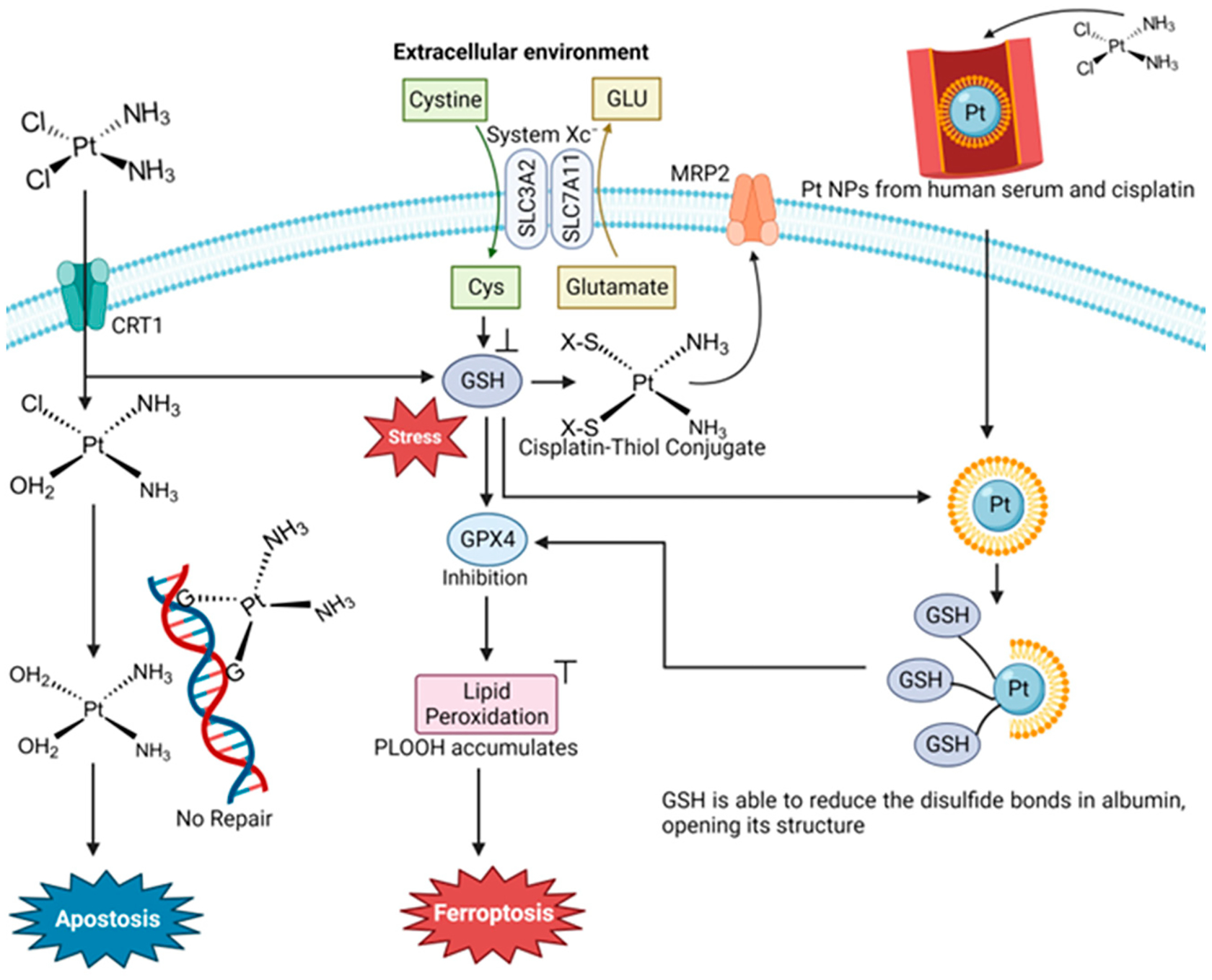
Proposed mechanisms of cisplatin’s induction of apoptosis and ferroptosis (image created using Biorender.com).

Due to an interest in tapping into molecular mechanisms that could trigger ferroptosis in cancer, Guo et al. examined the ferroptosis-inducing capability of traditional chemotherapy drugs in six different cancer cells [9]. They observed that cisplatin could induce ferroptosis as well as apoptosis in A549 non-small cell lung cancer (NSCLC) and HCT116 human colorectal cancer cells [9]. It was found that in these cells, Pt(II) significantly decreases the reduced form of glutathione (GSH) via direct coordination to the molecule (Figure 11). It is known that when cisplatin enters the cell, 60% of it binds to glutathione in the cytoplasm [86,87]. Typically, as already described, this complexation serves to decrease its cytotoxicity by effluxing the metal, but in these cell lines, the counter effect of ferroptosis appears to outcompete.

**Figure 12.**
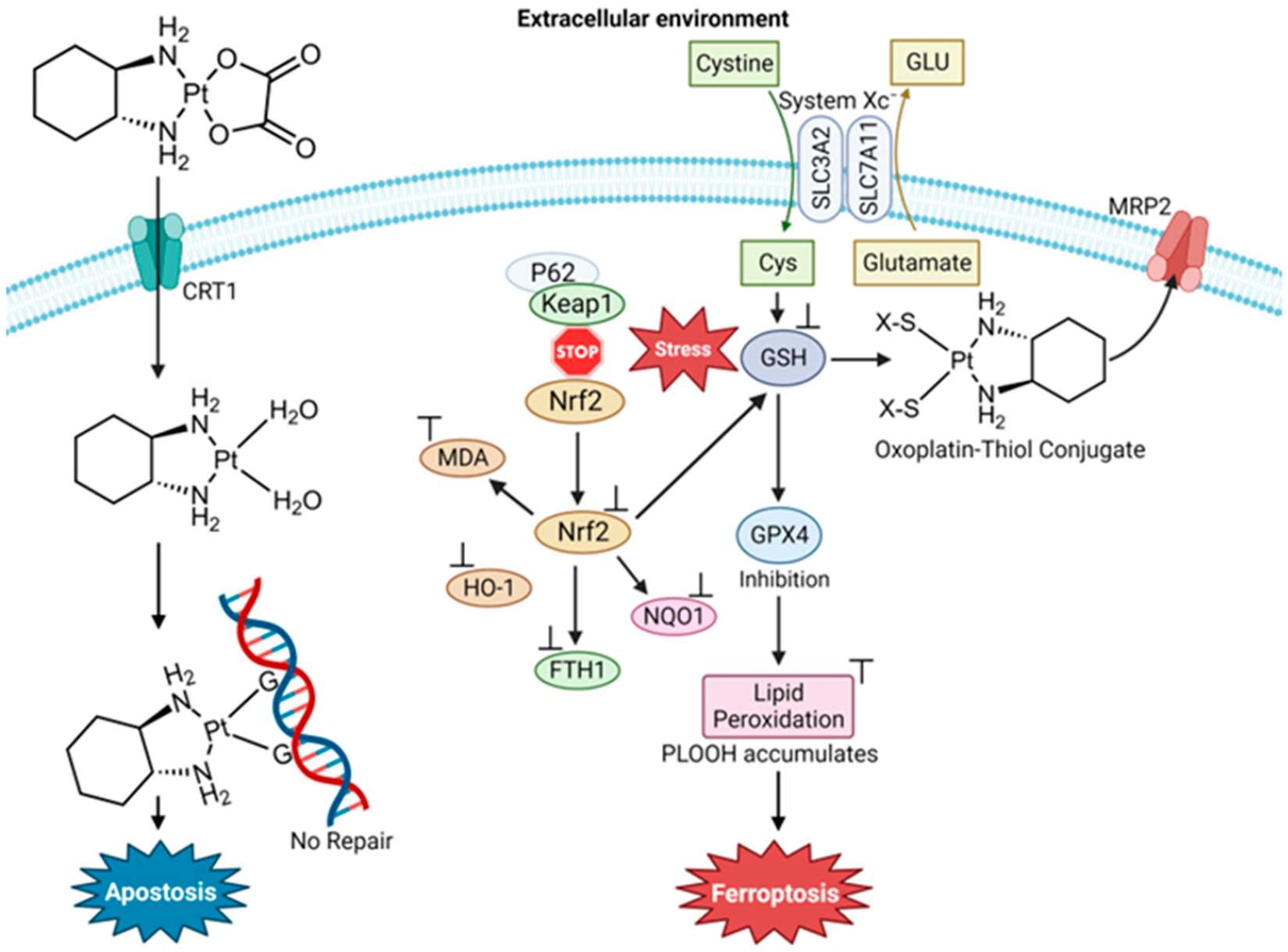
Proposed mechanism of oxaliplatin’s induction of apoptosis and ferroptosis (image created using Biorender.com) [89].

Oxaliplatin (Figure 12) was advanced to the drug market because of its improved safety profile compared to cisplatin and its lack of cross-resistance to cisplatin and carboplatin. The final cytotoxic metabolite of the drug retains the nonhydrolyzable diaminocyclohexane carrier ligand. Similar to cisplatin, oxaliplatin targets DNA by generating guanine cross-links [88]. Oxaliplatin is a first-line chemo drug for colorectal cancer (CCR). Liu et al. studied the effect of oxaliplatin in HT29 colorectal cancer cells to determine whether it could induce ferroptosis and oxidative stress for insight into new strategies for this highly aggressive cancer [89]. They found that oxaliplatin triggers ferroptosis by blocking the Nrf2 signaling pathway, resulting in the increase of MDA [89]. Very importantly, they observed that oxaliplatin enhances the ferroptosis-inducing effect of erastin on CRC cells [89].

A recent finding regarding Pt(II) drug blood speciation may also be attributed to the ferroptosis capability of these compounds [90]. Adult patients receive a standard 90 mg m^−2^ dosage of cisplatin. Blood isolated from adults following a round of chemotherapy was found to contain Pt NPs with an average diameter of 6–8 nm and a serum albumin corona of 30 nm in thickness. When the Pt NPs enter into K562 cells, the presence of the mM amount of cytosolic glutathione is capable of dismantling the protein corona, leaving behind the bare Pt NPs [90]. In this form, the Pt NPs serve as a GSH sponge as the thiol group anchors onto the Pt surface. This GSH anchoring seemingly prevents the GSH-induced efflux of the Pt while also making the GSH biounavailable (Figure 11).

Currently, efforts are directed at incorporating Pt(II) compounds into different types of anticancer strategies that aim to improve the cancer specificity of the Pt(II). Some strategies include those directed at the development of species that can easily be activated by light and lead to cell death; transition metals being particularly of interest in this area as different types of electronic transitions can be exploited, resulting in longer-term effects and more potent cytotoxicity. Chen et al. developed a degradable NP incorporating a biodegradable Fe(III)-polydopamine (FeP) core with photothermal capability and a hyaluronic acid (HA) crosslinked cisplatin (PtH) shell to achieve a synergistic multifunctional anticancer agent that operates by amplifying intracellular oxidative stress [91]. PtH@FeP NPs had a ratio of Pt:Fe ranging from 4:3 to 5:3, possibly due to the lack of a reproducible composition. The FeP core facilitated endosomal escape because of the “proton-sponge effect” [92]. The polydopamines are weakly basic and absorb free protons in the endosome, preventing them from escaping the endosome through diffusion and thus no longer contributing to the internal pH of the endosome. As the absorbed protons accumulate, they cause the membrane potential to increase past the equilibrium level, which results in an influx of chloride and an increase in osmotic pressure. The endosome swells until a critical point at which the lipid bilayer membrane ruptures and releases the endosome contents into the cell. The HA-coated shell enabled the NPs to actively target cancer cells which overexpress the HA receptor. The spherically shaped and negatively charged PtH@FeP NPs were 230 nm in diameter. They enlarge to 602.6 ± 15.2 nm at pH 6.5 attributed to the gelatinous HA shell, which is believed to facilitate long-term drug retention in tumor sites. UV-Vis and IR spectra exhibited the NIR light absorbance of the PtH@FeP NPs. Pt/Fe release from the NPs was investigated under conditions that replicate the extracellular matrix (pH 6.5) and intracellular-reducing environment (10 mM GSH pH 7.4) at 37 °C. Negligible release was observed at pH 6.5. More significant but slow release was observed after treatment with GSH. PtH@FeP showed Pt and Fe cumulative release of 61.43% and 30.21% after 72 h [91]. The metal release is postulated to be due to the GSH coordination of the Fe(III). It is expected that the PtH@FeP NPs facilitate cytotoxicity through PTT activation at 808 nm laser exposure followed by endosomal release of the NPs. GSH-triggered degradation of the NPs then leads to the released cisplatin attacking DNA and the released labile Fe undergoing Fenton reactivity and ferroptosis onset.

PtH@FeP NP cytotoxicity was examined against 4T1 (murine mammary carcinoma) and HepG2 (hepatocellular carcinoma) cells [91]. In the case of 4T1, treatment with the NP containing 40 μM Pt and 30 μM Fe and laser application resulted in the almost complete loss of viability after 24 h. In contrast, HepG2 exhibited a nearly 75% viability reduction. Additional characterizations were performed with the 4T1 cell line. Biomarker studies of the NP treatment of the cells showed signature ferroptosis and caspase-dependent apoptosis cell death characteristics. In vivo studies were performed with female Balb/c mice containing 4T1 tumors. The mice were separated into five groups and administered different treatments: PBS, cisplatin, PtH (Pt at 5 mg/kg), PtH@FeP with or without laser (Pt at 3 mg/kg and Fe at 1.8 mg/kg). After 24 h, the tumors were exposed to laser excitation at 808 nm (1 W) for 1 min. Throughout the experiment, the mice’s body weight and tumor volume were closely monitored for 21 days. Cisplatin treatment inhibited tumor growth and even caused a decrease in tumor size with respect to the buffer control, but none of the mice survived after 10 days due to the compound’s toxicity. PtH did not lower the relative tumor volume to the same extent as free cisplatin but it did significantly reduce the compound’s toxicity. There was no significant difference in relative tumor volume between the PtH and PtH@FeP treatment. However, upon irradiation of PtH@FeP, complete suppression of the tumor was observed owing to the synergism of the PTT effect, without any mice’s demise [91]. Organ histopathology analysis was performed using hematoxylin and eosin (H&E) staining and no organ damage was observed, indicative of the cancer specificity of the system.

### 4.2. Auranofin

Auranofin (AF) was first designed as a therapy for rheumatoid arthritis (RA) and was FDA-approved for this purpose in 1985 [93,94]. Interest in the compound as an anticancer agent stemmed from the decline in cancer progression in certain RA patients being treated with AF. It is a linear Au(I) compound, in which the Au(I) is coordinated to a trialkylphosphine and an S-glycosyl group (2,3,4,6-tetra-*O*-acetyl-1-thio-β-d-glucopyranose). The compound is believed to operate as a prodrug like cisplatin [95] and is capable of inducing cell death in cisplatin-resistant cells [96]. The sugar group dissociates rapidly and the Au-PEt_3_^+^ complex enters into the cytosol [97]. Unlike Pt(II) from the Pt(II) drugs, DNA is not the primary target of Au(I). The antiproliferative behavior is believed to be due to the inhibition of thioredoxin (Trx) and thioredoxin reductase (TrxR), which are responsible for regulating redox processes and helping to maintain cellular iron homeostasis [98]. Au(I) inhibition of the Trx/TrxR system results from binding to their active site thiol and seleno residues [99,100]. This interaction results in a change in the redox regulation within cells, leading to the excess production of ROS [99,100]. High ROS levels disturb the activity of cytosolic peroxiredoxin enzymes (Prxs) and activate p38 MAP Kinase (MAPK), which then leads to the initiator caspase triggering the onset of apoptosis. The disturbance of Prxs results in mitochondrial damage and permeability and the release of cytochrome c, turning on its apoptotic function [100].

**Figure 13.**
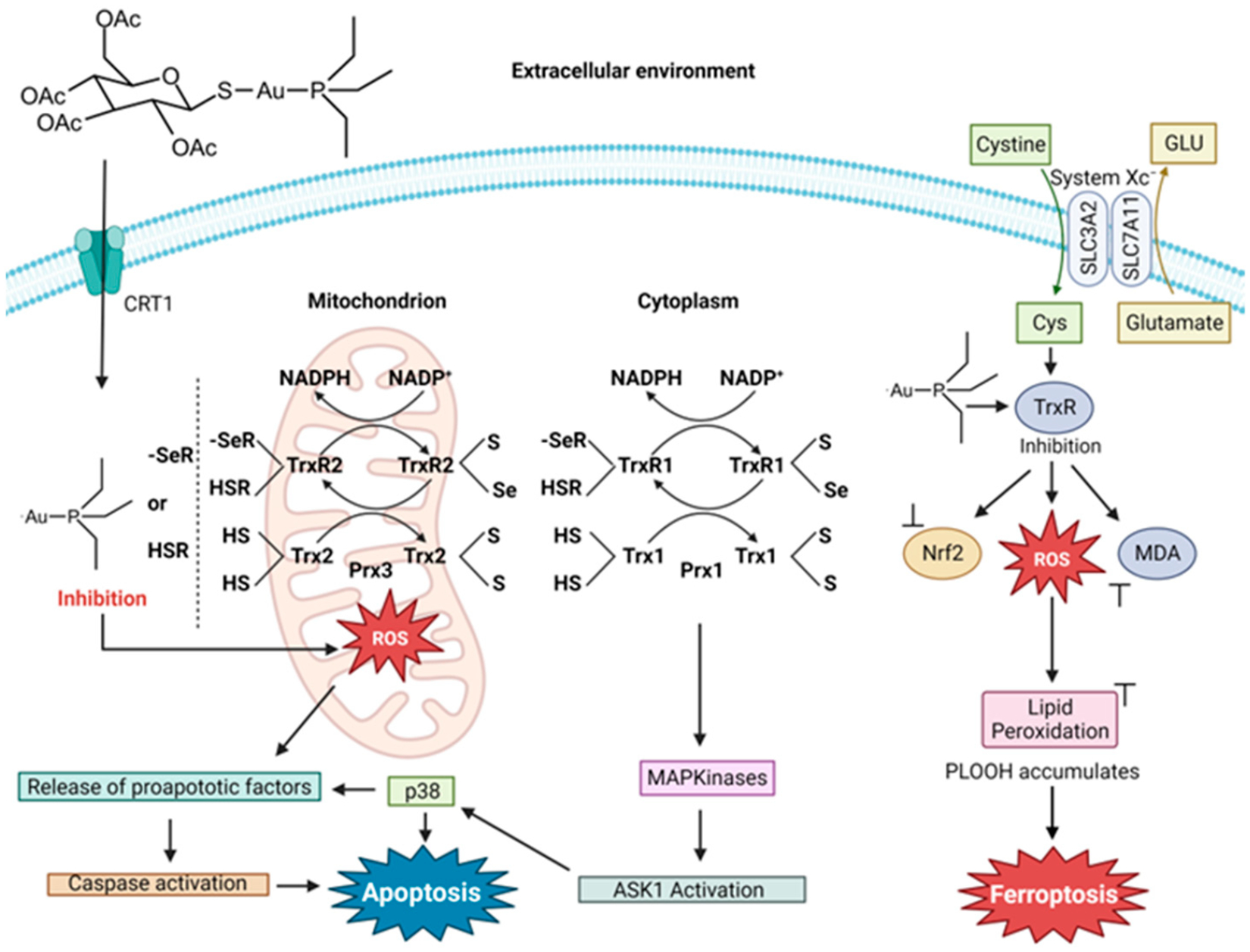
Proposed mechanism of auranofin’s induction of apoptosis and ferroptosis (image created using Biorender.com).

The peptide hormone hepcidin, like the Trx/TrxR system, participates in maintaining cellular iron/redox homeostasis [98]. Hepcidin deficiency can induce iron overload diseases such as hereditary hemochromatosis and anemia. Yang et al. sought to study iron modulation by screening for hepcidin agonists in human hepatic Huh7 cells using a library of over 640 FDA-approved drugs [11]. They found that AF activates IL-6 signaling, which is responsible for upregulating hepcidin expression. The activation was through the NF-κB pathway. They validated these results in C57BL/6J mice and a mouse model of hemochromatosis (Hfe^−/−^ mice). Acute treatment of C57BL/6J mice with 5 mg/kg AF decreased the serum Fe by way of activated hepcidin signaling, while chronic treatment of Hfe^−/−^ mice with 5 mg/kg AF decreased the systemic iron overload in male mice but less so in female mice. Contrary to this beneficial effect, Yang et al. observed that AF is able to induce ferroptosis by way of the inhibition of TrxR activity. Several biomarkers of ferroptosis were detected, including lipid peroxidation. The use of the ferroptosis inhibitor Fer-1 could inhibit the appearance of these biomarkers. In C57BL/6J mice, the role of TrxR inhibition with respect to ferroptosis was revealed through the administration of TRi-1 (25 mg/kg), a specific TrxR inhibitor. Decreased TrxR activity resulted in an accumulation of hepatic lipid peroxidation. By attenuating the ferroptosis capability of AF via the use of Fer-1, AF could exhibit the capacity to decrease the systemic Fe levels. This work reveals how Au(I) activity can be finetuned to turn on and off its ferroptosis induction. For an Fe-based anticancer strategy, turning on Au(I) ferroptosis induction would be very useful (Figure 13).

Deben et al. sought a deeper understanding of the anticancer mechanism of action of AF and, in particular, its potential application against NSCLC [101]. This cancer type has a significant elevation of TrxR activity in vitro and in vivo [102]. Interestingly, high levels of TrxR are associated with chemotherapy resistance, including cisplatin [103]. Mutant forms of the tumor suppressor protein p53 were found to be sensitizers of AF treatment. Mutant p53 has the potential to alter the cellular redox balance by preventing activation and NF-E2-related factor 2 (NRF2) function. As the main regulator of antioxidant transcription, its inhibition generates an excess of ROS within cancer cells [104]. Deben et al. worked with NCI-H1299 (p53 null) and its two isogenic derivatives with the accumulation of mutant p53 R175H or R273H. They found that the main mechanism of AF, at both cytostatic (1 μM) and cytotoxic (5 μM) concentrations, is the inhibition of TrxR. The method of cell death induced by AF is dependent on the p53 mutant type. AF sensitized mutant p53 R175H NSCLC cells to caspase-3/7-dependent apoptosis, whereas p53 R273H cells were more vulnerable to ferroptosis [101]. Regardless of the method of cell death, at a 5 μM concentration, AF depletes GSH. In a study with eight NSCLC and pancreatic ductal adenocarcinoma (PDAC) cell lines with differing p53 status, AF more effectively killed mutant p53 cells with higher p53 protein levels [101].

### 4.3. Photochemotherapeutic Metallodrugs

The practical application of therapeutic treatments for hypoxic and refractory solid tumors has been hindered by their limited efficacy. Photodynamic therapy (PDT) is a treatment option that utilizes a photosensitizing agent and light source to eradicate cancer cells. The utilization of ROS and precise photoirradiation has shown great potential in the struggle against hard-to-treat tumors, resulting in minimal resistance and limited invasiveness. Although ROS cause imprecise damage, the precise photoirradiation allows for a targeted approach. Additionally, PDT offers a diverse range of cell death mechanisms [105,106].

**Figure 14.**
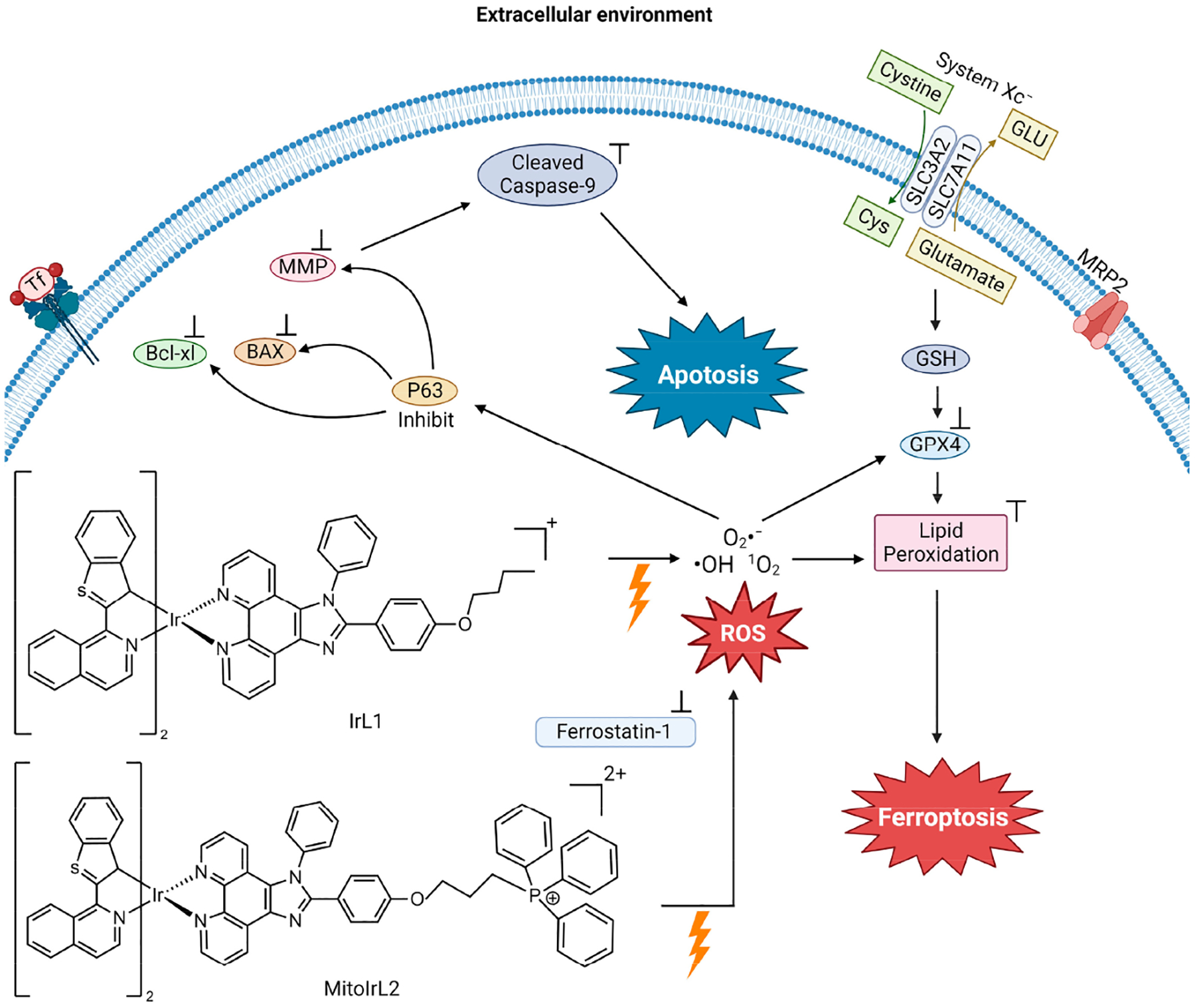
Proposed mechanism of IrL1 and MitoIrL2’s induction of apoptosis and ferroptosis (image created using Biorender.com) [107].

Hao et al. synthesized two Ir(III) complexes with a coordination number 6, containing two 1-benzo[b]thien-2-yl-isoquinoline (btiq) ligands and an imidazophenanthroline (ipt) auxiliary ligand; IrL1 and MitoIrL2 (Figure 14) to examine their use as PDT agents for hypoxic cells [107]. Both complexes exhibit intensive absorption bands at ~488 nm due to a metal-to-ligand charge transfer (MLCT). The complexes display a near-IR emission at ~685 nm, with a shoulder at 740 nm. The emission intensity decreases in the presence of ambient O_2_, indicative of excited forms of the compounds possibly being able to undergo intersystem crossing (ISC) to react with O_2_ and produce ROS. Given these physicochemical properties, the complexes were studied as photodynamic therapy (PDT) agents. They analyzed the ROS species formed in Michigan Cancer Foundation-7 (MCF-7) breast cancer cells in two different biological environments. These are normoxia (an environment with normal levels of oxygen) and hypoxia (an environment with a reduction in oxygen) because they expected that these compounds would be able to overcome the resistance of cells in a hypoxic environment. The IrL1 and MitoIrL2 complexes at 5 μM were photoirradiated at 450 nm (30 Jcm^−2^). In a normoxia environment, they produce singlet oxygen (^1^O_2_), but not in the hypoxic environment. An increase in the ROS species O_2_^−^ and ·OH were observed under both conditions, but MitoIrL2 produced a greater amount [107]. At a concentration of 1 μM of both complexes, the GPX4 levels decreased and lipid peroxide accumulated [107].

The mechanism of cell death was determined by incorporating the inhibitors of apoptosis (z-VADfmk), necrosis (necrostatin-1, Nec-1), autophagy (3-methyladenine, 3-MA) and ferroptosis (Fer-1) under hypoxic conditions in a cell viability assay [107]. Fer-1 addition produced an increase in cell viability in treatments with both complexes, indicating that both induce ferroptosis. The z-VADfmk inhibitor increased the cell viability in treatment with MitoIrL2, suggesting that the compound also induces apoptosis. For this compound, apoptosis is the dominant cell death mechanism. According to flow cytometry, IrL1 caused apoptotic cell death in 9.5% of the total dead cells, whereas MitoIrL2 caused 43.1%. Western blot assays were carried out to assess apoptosis biomarkers. MitoIrL2 increased the p53 and Bax levels, while the Bcl-xl levels decreased, leading to a rise in caspase-9 [107]. The compound was observed to inhibit ATP production and cause mitochondrial structural changes. This study elucidates how the finetuning of the physicochemical properties of a PDT agent can increase the cytotoxic potency of a metal compound and facilitate dual cell death mechanisms.

**Figure 15.**
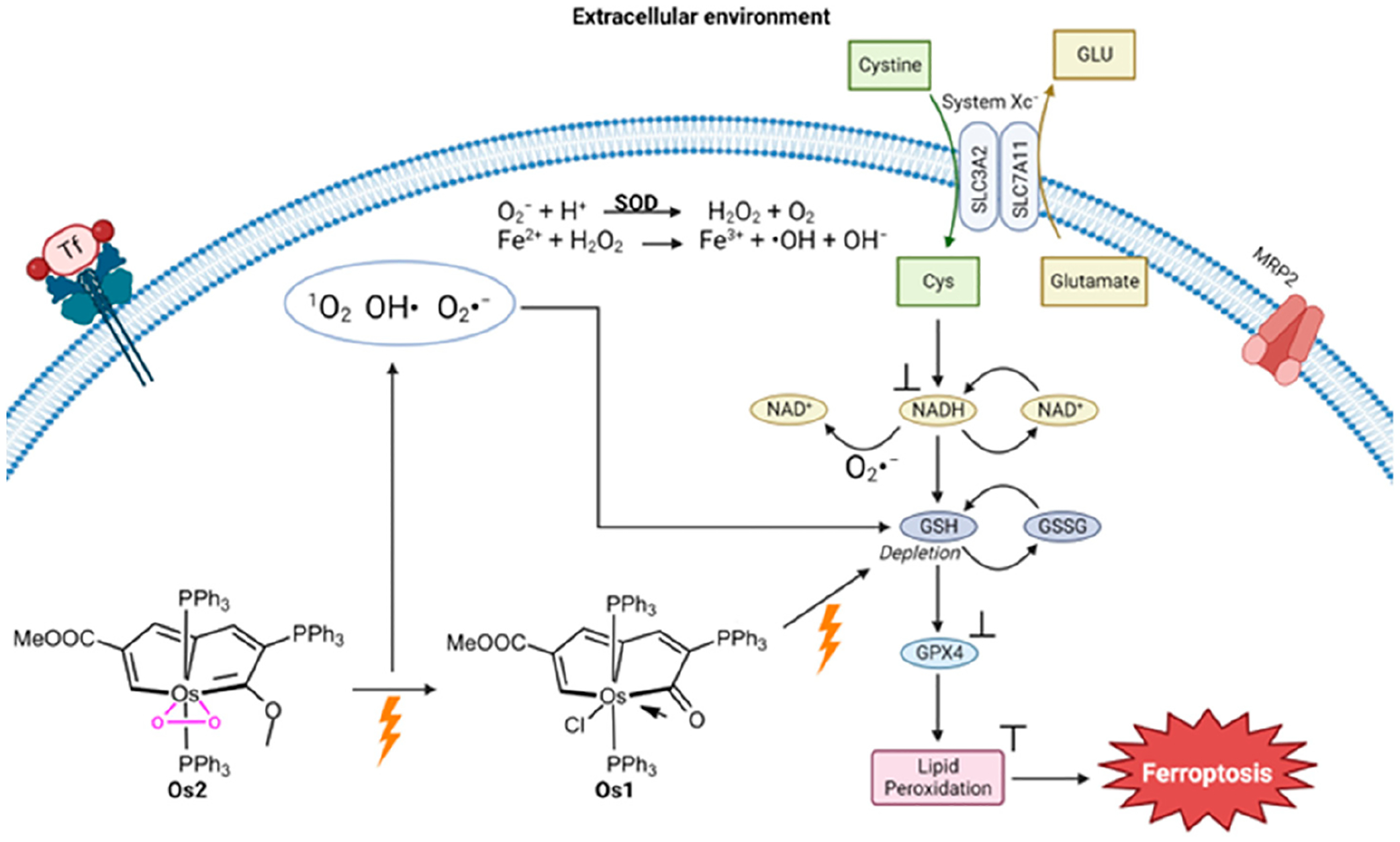
Proposed mechanism of Os2’s induction of ferroptosis (image created using Biorender.com) [108].

Another metal that has been studied for its ability to trigger ferroptosis via photodynamic therapy (PDT) is osmium. Zhang et al. investigated a way to treat hypoxic cancer cells using a pentagonal bipyramidal complex of Os (Os2) containing pentalyne and peroxo ligands in the equatorial plane and two triphenylphosphine ligands in the axial position [108]. Using photoirradiation under light illumination (465 nm, 13 mW/cm^2^), Os2 releases its peroxo ligand and converts it into Os1 (Figure 15) and the superoxide (O_2_^−^) ROS. An ROS detection experiment was performed with HeLa cervical cancer cells treated with Os2 under hypoxic conditions and light irradiation. The generation of O_2_^−^ was observed as well as OH and ^1^O_2_. Under normal oxygen conditions, exposure to Os2 and Os1 results in considerable cell death following irradiation, with the viability of HeLa cells dropping below 20%. Similar results are observed in hypoxia, although the degree of cell death is reduced at lower concentrations, both before and after irradiation, compared to normoxia. After administering Os2 treatment, the GSH percentage in cells was examined and the results showed that the GSH levels in the irradiated group were significantly lower than those in the non-irradiated group. The relative expression levels of GPX4 were lower post-irradiation, and in fact, were at the similar level as HeLa cell treatment with the ferroptosis inducer RSL3. The photocatalytic oxidation of NADH into NAD+ was observed by Os2 under light irradiation, which is characteristic of ferroptosis induction.

An in vivo study was conducted by treating Balb/c mice implanted with HeLa tumors [108]. Following 7 days after cell injection, mice with a desired tumor volume (~100 mm^3^) were selected for further study. The mice were separated into four groups: control dark group with only PBS injection, control light irradiation group, Os2-dark group, and Os2-light irradiation group. The irradiation (465 nm with an intensity of 13 mW/cm^2^ for a duration of 60 min) was performed on day zero and the tumor size was recorded every two days. It was observed that the Os2-light irradiation group had the lowest growth in tumor size, roughly 2.33 times less growth than the control dark group. Biosafety analysis of the Os compound was performed by injecting healthy mice with three times the therapeutic dose and performing H&E staining of thin slices of various organs. No tissue damage was observed. Toxicity was also examined in zebrafish and no blood vessel damage was detected. These results attest to the cancer specificity and low toxicity of the Os2 system.

## 5. Conclusions

This assessment of various anticancer strategies that induce ferroptosis reveals key features for drug design to effectively disrupt the Fe balance and the corresponding regulation of lipid peroxidation. Metal chelators can contribute to offsetting this balance in different ways. Triapine and analogues, for instance, demonstrate how high affinity Fe(II/III) chelation can deplete the bioavailability of Fe within cells and transform the metal into highly toxic agents through redox cycling. Other chelators perturb the Fe balance by serving as ionophores of Cu and Zn, resulting in excessive levels of these metals. Cu can be directed to the mitochondria and induce damage due to the redox activity of the metal and the uncontrolled generation of ROS. Zn(II) can replace Fe at important biomolecular sites and attenuate Fe-based functionality due to being redox inert. Thus, excess Cu and Zn levels alter the antioxidant defenses. Cu chelators that deplete intracellular Cu also yield an Fe imbalance by inhibiting the redox regulation that Cu imposes on Fe and that is responsible for the proper biodistribution of Fe. In the same vein, nanoparticles based on Cu(II) and Zn(II) can function to exploit the redox activity of Cu and to deliver a high payload of Zn(II) release into cells, respectively.

Small molecule non-iron metal complexes demonstrate both the importance of the metal center and the physicochemical properties of intact metal–ligand adducts. The classical Pt(II) and Au(I) drugs reveal how soft Lewis acid metal ions can strongly bind to thiol and seleno proteins and ablate redox regulation, leading to ferroptosis. In the case of Au(I), the particularities of the tumor biochemical environment, such as the presence of particular mutant P53 proteins, dictate whether Au(I) is able to turn on ferroptosis [101]. Some metal complexes that are light sensitive demonstrate photosensitizing and photothermal capability to facilitate tumor-localized applications that can be coupled with ferroptosis-inducing agents. Photosensitizing metal complexes demonstrate the utility of PDT to enable the generation of ROS and ^1^O_2_ that can synergize with intracellular Fe to produce LPO. PTT agents can be synergized with redox active metals to facilitate more than one type of death.

The use of drug carriers such as nanosystems can enable taking advantage of the EPR effect to target tumors. However, the reality is that only about 5% of an administered NP drug typically ends up in the tumor and thus selectivity is not greatly enhanced [85]. Coating NPs with functional groups that target receptors overexpressed on cancer cells can help to overcome this limitation, as seen in examples included in this study. While NPs allow the shuttling of more than one cancer agent to facilitate combined anticancer strategies, the complex nature of the composition required to bind these agents and also possess receptor targeting functional groups can result in hard to reproduce structures, as highlighted in the case of the PtH@FeP NPs [91].

Collectively, these studies reveal that it is highly unlikely that any drug strategy can purely induce ferroptosis alone, which is not a bad thing. The synergism of approaches to trigger multiple cell death pathways may prove vital for the drug development of far more potent and less cancer-resistant chemotherapy.

## References

[1] Nie Q; Hu Y; Yu X; Li X; Fang X Induction and application of ferroptosis in cancer therapy. Cancer Cell Int. 2022, 22, 12. 34996454 10.1186/s12935-021-02366-0 PMC8742449

[2] Debela DT; Muzazu SG; Heraro KD; Ndalama MT; Mesele BW; Haile DC; Kitui SK; Manyazewal T New approaches and procedures for cancer treatment: Current perspectives. SAGE Open Med. 2021, 9, 20503121211034366. 34408877 10.1177/20503121211034366 PMC8366192

[3] Peng F; Liao M; Qin R; Zhu S; Peng C; Fu L; Chen Y; Han B Regulated cell death (RCD) in cancer: Key pathways and targeted therapies. Signal Transduct. Target Ther 2022, 7, 286. 35963853 10.1038/s41392-022-01110-y PMC9376115

[4] Schulze-Osthoff K; Ferrari D; Los M; Wesselborg S; Peter ME Apoptosis signaling by death receptors. Eur. J. Biochem 1998, 254, 439–459. 9688254 10.1046/j.1432-1327.1998.2540439.x

[5] Dixon SJ; Lemberg KM; Lamprecht MR; Skouta R; Zaitsev EM; Gleason CE; Patel DN; Bauer AJ; Cantley AM; Yang WS; Ferroptosis: An iron-dependent form of nonapoptotic cell death. Cell 2012, 149, 1060–1072. 22632970 10.1016/j.cell.2012.03.042 PMC3367386

[6] Bannai S; Kitamura E Transport interaction of L-cystine and L-glutamate in human diploid fibroblasts in culture. J. Biol. Chem 1980, 255, 2372–2376. 7358676

[7] Dolma S; Lessnick SL; Hahn WC; Stockwell BR Identification of genotype-selective antitumor agents using synthetic lethal chemical screening in engineered human tumor cells. Cancer Cell 2003, 3, 285–296. 12676586 10.1016/s1535-6108(03)00050-3

[8] Yang WS; SriRamaratnam R; Welsch ME; Shimada K; Skouta R; Viswanathan VS; Cheah JH; Clemons PA; Shamji AF; Clish CB; Regulation of ferroptotic cancer cell death by GPX4. Cell 2014, 156, 317–331. 24439385 10.1016/j.cell.2013.12.010 PMC4076414

[9] Guo J; Xu B; Han Q; Zhou H; Xia Y; Gong C; Dai X; Li Z; Wu G Ferroptosis: A novel anti-tumor action for cisplatin. Cancer Res. Treat 2018, 50, 445–460. 28494534 10.4143/crt.2016.572 PMC5912137

[10] Palmer LD; Jordan AT; Maloney KN; Farrow MA; Gutierrez DB; Gant-Branum R; Burns WJ; Romer CE; Tsui T; Allen JL; Zinc intoxication induces ferroptosis in A549 human lung cells. Metallomics 2019, 11, 982–993. 30968088 10.1039/c8mt00360b PMC6531343

[11] Yang L; Wang H; Yang X; Wu Q; An P; Jin X; Liu W; Huang X; Li Y; Yan S; Auranofin mitigates systemic iron overload and induces ferroptosis via distinct mechanisms. Signal Transduct. Target Ther 2020, 5, 138. 32732975 10.1038/s41392-020-00253-0 PMC7393508

[12] Yang WS; Kim KJ; Gaschler MM; Patel M; Shchepinov MS; Stockwell BR Peroxidation of polyunsaturated fatty acids by lipoxygenases drives ferroptosis. Proc. Natl. Acad. Sci. USA 2016, 113, E4966–E4975. 27506793 10.1073/pnas.1603244113 PMC5003261

[13] Zhang P; Liu C; Wu W; Mao Y; Qin Y; Hu J; Hu J; Fu J; Hua D; Yin J Triapine/Ce6-loaded and lactose-decorated nanomicelles provide an effective chemo-photodynamic therapy for hepatocellular carcinoma through a reactive oxygen species-boosting and ferroptosis-inducing mechanism. Chem. Eng. J 2021, 425, 131543.

[14] Gao W; Huang Z; Duan J; Nice EC; Lin J; Huang C Elesclomol induces copper-dependent ferroptosis in colorectal cancer cells via degradation of ATP7A. Mol. Oncol 2021, 15, 3527–3544. 34390123 10.1002/1878-0261.13079 PMC8637554

[15] Luo Y; Huang Q; He B; Liu Y; Huang S; Xiao J Regulation of ferroptosis by non-coding RNAs in the development and treatment of cancer (Review). Oncol. Rep 2021, 45, 29–48. 10.3892/or.2020.7836 PMC7709825 33155665

[16] Wu W; Geng Z; Bai H; Liu T; Zhang B Ammonium Ferric Citrate induced Ferroptosis in Non-Small-Cell Lung Carcinoma through the inhibition of GPX4-GSS/GSR-GGT axis activity. Int. J. Med. Sci 2021, 18, 1899–1909. 33746607 10.7150/ijms.54860 PMC7976582

[17] Lewerenz J; Hewett SJ; Huang Y; Lambros M; Gout PW; Kalivas PW; Massie A; Smolders I; Methner A; Pergande M; The cystine/glutamate antiporter system x(c)(−) in health and disease: From molecular mechanisms to novel therapeutic opportunities. Antioxid. Redox Signal 2013, 18, 522–555. 22667998 10.1089/ars.2011.4391 PMC3545354

[18] Dixon SJ; Stockwell BR The Hallmarks of Ferroptosis. Ann. Rev. Cancer Biol 2019, 3, 35–54. 10.1146/annurev-cancerbio-030518-055844 PMC12851585 41613499

[19] Shaghaghi Z; Motieian S; Alvandi M; Yazdi A; Asadzadeh B; Farzipour S; Abbasi S Ferroptosis Inhibitors as Potential New Therapeutic Targets for Cardiovascular Disease. Mini Rev. Med. Chem 2022, 22, 2271–2286. 35184711 10.2174/1389557522666220218123404

[20] Zheng J; Conrad M The Metabolic Underpinnings of Ferroptosis. Cell Metab. 2020, 32, 920–937. 33217331 10.1016/j.cmet.2020.10.011

[21] Liang X; Chen M; Bhattarai P; Hameed S; Tang Y; Dai Z Complementing Cancer Photodynamic Therapy with Ferroptosis through Iron Oxide Loaded Porphyrin-Grafted Lipid Nanoparticles. ACS Nano 2021, 15, 20164–20180. 34898184 10.1021/acsnano.1c08108

[22] Casas Fernandez JS; Moreno Martinez V; Sanchez Gonzalez A; Sanchez Lopez JL; Sordo Rodriguez J Quimica Bioinorganica; Editorial Sintesis: Madrid, Spain, 2002.

[23] Mannargudi MB; Deb S Clinical pharmacology and clinical trials of ribonucleotide reductase inhibitors: Is it a viable cancer therapy? J. Cancer Res. Clin. Oncol 2017, 143, 1499–1529. 28624910 10.1007/s00432-017-2457-8 PMC11819458

[24] Greene BL; Kang G; Cui C; Bennati M; Nocera DG; Drennan CL; Stubbe J Ribonucleotide reductases: Structure, chemistry, and metabolism suggest new therapeutic targets. Annu. Rev. Biochem 2020, 89, 45–75. 32569524 10.1146/annurev-biochem-013118-111843 PMC7316142

[25] Agarwal D; Goodison S; Nicholson B; Tarin D; Urquidi V Expression of matrix metalloproteinase 8 (MMP-8) and tyrosinase-related protein-1 (TYRP-1) correlates with the absence of metastasis in an isogenic human breast cancer model. Differentiation 2003, 71, 114–125. 12641565 10.1046/j.1432-0436.2003.710202.x

[26] Shen Y; Li X; Dong D; Zhang B; Xue Y; Shang P Transferrin receptor 1 in cancer: A new sight for cancer therapy. Am. J. Cancer Res 2018, 8, 916–931. 30034931 PMC6048407

[27] Chen Y; Fan Z; Yang Y; Gu C Iron metabolism and its contribution to cancer (Review). Int. J. Oncol 2019, 54, 1143–1154. 30968149 10.3892/ijo.2019.4720

[28] Bystrom LM; Rivella S Cancer cells with irons in the fire. Free Radic. Biol. Med 2015, 79, 337–342. 24835768 10.1016/j.freeradbiomed.2014.04.035 PMC4232494

[29] Lane DJ; Merlot AM; Huang ML; Bae DH; Jansson PJ; Sahni S; Kalinowski DS; Richardson DR Cellular iron uptake, trafficking and metabolism: Key molecules and mechanisms and their roles in disease. Biochim. Biophys. Acta 2015, 1853, 1130–1144. 25661197 10.1016/j.bbamcr.2015.01.021

[30] Bertini I; Gray HB; Stiefel EI; Valentine JS Biological Inorganic Chemistry: Structure and Reactivity; University Science Books: New York, NY, USA, 2007.

[31] Chen X; Yu C; Kang R; Tang D Iron metabolism in ferroptosis. Front. Cell Dev. Biol 2020, 8. 10.3389/fcell.2020.590226 PMC7575751 33117818

[32] Grignano E; Birsen R; Chapuis N; Bouscary D From iron chelation to overload as a therapeutic strategy to induce ferroptosis in leukemic cells. Front. Oncol 2020, 10. 10.3389/fonc.2020.586530 PMC7530268 33042852

[33] Li J; Zhang W From iron chelation to overload as a therapeutic strategy to induce ferroptosis in hematologic malignancies. Hematology 2022, 27, 1163–1170. 36222350 10.1080/16078454.2022.2132362

[34] Wu LY; Song ZY; Li QH; Mou LJ; Yu YY; Shen SS; Song XX Iron chelators reverse organ damage in type 4B hereditary hemochromatosis. Medicine 2021, 100, e25258. 33787609 10.1097/MD.0000000000025258 PMC8021318

[35] Soliman AT The Effects of Treatment with Blood Transfusion, Iron Chelation and Hydroxyurea on Puberty, Growth and Spermatogenesis in Sickle Cell Disease (SCD): A short update. Acta Biomed. 2021, 92, 1–8. 10.23750/abm.v92i4.11917 PMC8477091 34487059

[36] Rayatpour A; Foolad F; Heibatollahi M; Khajeh K; Javan M Ferroptosis inhibition by deferiprone, attenuates myelin damage and promotes neuroprotection in demyelinated optic nerve. Sci. Rep 2022, 12, 19630. 36385152 10.1038/s41598-022-24152-2 PMC9668997

[37] Yao X Deferoxamine promotes recovery of traumatic spinal cord injury by inhibiting ferroptosis. Neural Regen. Res 2019, 14, 532–541. 30539824 10.4103/1673-5374.245480 PMC6334606

[38] Jomen W Iron chelator deferasirox inhibits NF-κB activity in hepatoma cells and changes sorafenib-induced programmed cell deaths. Biomed. Pharmacother 2022, 153, 1–12. 10.1016/j.biopha.2022.113363 35834989

[39] Ryan SK; Ugalde CL; Rolland AS; Skidmore J; Devos D; Hammond TR Therapeutic inhibition of ferroptosis in neurodegenerative disease. Trends Pharmacol. Sci 2023, 44, 674–688. 37657967 10.1016/j.tips.2023.07.007

[40] Fratta Pasini AM; Stranieri C; Busti F; Di Leo EG; Girelli D; Cominacini L New Insights into the Role of Ferroptosis in Cardiovascular Diseases. Cells 2023, 12, 867. 36980208 10.3390/cells12060867 PMC10047059

[41] Chen Y; Guo X; Zeng Y; Mo X; Hong S; He H; Li J; Fatima S; Liu Q Oxidative stress induces mitochondrial iron overload and ferroptotic cell death. Sci. Rep 2023, 13, 15515. 37726294 10.1038/s41598-023-42760-4 PMC10509277

[42] Enyedy ÉA Complex formation and cytotoxicity of Triapine derivatives: A comparative solution study on the effect of the chalcogen atom and NH-methylation. Dalton Trans. 2020, 49, 16887–16902. 33185224 10.1039/d0dt03465g

[43] Enyedy EA; Primik MF; Kowol CR; Arion VB; Kiss T; Keppler BK Interaction of triapine and related thiosemicarbazones with iron(III)/(II) and gallium(III): A comparative solution equilibrium study. Dalton Trans. 2011, 40, 5895–5905. 21523301 10.1039/c0dt01835j PMC3372890

[44] Aye Y; Long MJC; Stubbe J Mechanistic Studies of Semicarbazone Triapine Targeting Human Ribonucleotide Reductase in Vitro and in Mammalian Cells. J. Biol. Chem 2012, 287, 35768–35778. 22915594 10.1074/jbc.M112.396911 PMC3471726

[45] Plamthottam S; Sun D; Van Valkenburgh J; Valenzuela J; Ruehle B; Steele D; Poddar S; Marshalik M; Hernandez S; Radu CG; Activity and electrochemical properties: Iron complexes of the anticancer drug triapine and its analogs. J. Biol. Inorg. Chem 2019, 24, 621–632. 31250199 10.1007/s00775-019-01675-0 PMC7849610

[46] Schweigel-Röntgen M Chapter Nine—The Families of Zinc (SLC30 and SLC39) and Copper (SLC31) Transporters. In Current Topics in Membranes; Bevensee MO, Ed.; Academic Press: Cambridge, MA, USA, 2014; Volume 73, pp. 321–355. 24745988 10.1016/B978-0-12-800223-0.00009-8

[47] Shi W; Zhang H; Zhang Y; Lu L; Zhou Q; Wang Y; Pu Y; Yin L Co-exposure to Fe, Zn, and Cu induced neuronal ferroptosis with associated lipid metabolism disorder via the ERK/cPLA2/AA pathway. Environ. Pollut 2023, 336, 122438. 37625769 10.1016/j.envpol.2023.122438

[48] Chen Z; Jiang R; Chen M; Zheng J; Chen M; Braidy N; Liu S; Liu G; Maimaitiming Z; Shen T; Multi-copper ferroxidase deficiency leads to iron accumulation and oxidative damage in astrocytes and oligodendrocytes. Sci Rep 2019, 9, 9437. 31263155 10.1038/s41598-019-46019-9 PMC6603037

[49] Kondaiah P; Yaduvanshi PS; Sharp PA; Pullakhandam R Iron and Zinc Homeostasis and Interactions: Does Enteric Zinc Excretion Cross-Talk with Intestinal Iron Absorption? Nutrients 2019, 11, 1885. 31412634 10.3390/nu11081885 PMC6722515

[50] Li F; Wu X; Liu H; Liu M; Yue Z; Wu Z; Liu L; Li F Copper Depletion Strongly Enhances Ferroptosis via Mitochondrial Perturbation and Reduction in Antioxidative Mechanisms. Antioxidants 2022, 11, 2084. 36358457 10.3390/antiox11112084 PMC9687009

[51] Collins JF; Prohaska JR; Knutson MD Metabolic crossroads of iron and copper. Nutr. Rev 2010, 68, 133–147. 20384844 10.1111/j.1753-4887.2010.00271.x PMC3690345

[52] La Fontaine S; Ackland ML; Mercer JF Mammalian copper-transporting P-type ATPases, ATP7A and ATP7B: Emerging roles. Int. J. Biochem. Cell Biol 2010, 42, 206–209. 19922814 10.1016/j.biocel.2009.11.007 PMC2846448

[53] Myint ZW; Oo TH; Thein KZ; Tun AM; Saeed H Copper deficiency anemia: Review article. Ann. Hematol 2018, 97, 1527–1534. 29959467 10.1007/s00277-018-3407-5

[54] Chen H; Attieh Z; Su T; Syed B; Gao H; Alaeddine R; Fox T; Usta J; Naylor C; Evans R; Hephaestin is a ferroxidase that maintains partial activity in sex-linked anemia mice. Blood 2004, 103, 3933–3939. 14751926 10.1182/blood-2003-09-3139

[55] Xue Q; Yan D; Chen X; Li X; Kang R; Klionsky DJ; Kroemer G; Chen X; Tang D; Liu J Copper-dependent autophagic degradation of GPX4 drives ferroptosis. Autophagy 2023, 19, 1982–1996. 36622894 10.1080/15548627.2023.2165323 PMC10283421

[56] Kannappan V; Ali M; Small B; Rajendran G; Elzhenni S; Taj H; Wang W; Dou QP Recent Advances in Repurposing Disulfiram and Disulfiram Derivatives as Copper-Dependent Anticancer Agents. Front. Mol. Biosci 2021, 8, 741316. 34604310 10.3389/fmolb.2021.741316 PMC8484884

[57] Ren X; Li Y; Zhou Y; Hu W; Yang C; Jing Q; Zhou C; Wang X; Hu J; Wang L; Overcoming the compensatory elevation of NRF2 renders hepatocellular carcinoma cells more vulnerable to disulfiram/copper-induced ferroptosis. Redox. Biol 2021, 46, 102122. 34482117 10.1016/j.redox.2021.102122 PMC8416961

[58] Bin B-H; Seo J; Kim ST Function, Structure, and Transport Aspects of ZIP and ZnT Zinc Transporters in Immune Cells. J. Immunol. Res 2018, 2018, 9365747. 30370308 10.1155/2018/9365747 PMC6189677

[59] Andreini C; Banci L; Bertini I; Rosato A Counting the zinc-proteins encoded in the human genome. J. Proteome Res 2006, 5, 196–201. 16396512 10.1021/pr050361j

[60] McClung JP Iron, Zinc, and Physical Performance. Biol. Trace Elem. Res 2019, 188, 135–139. 30112658 10.1007/s12011-018-1479-7

[61] Yamasaki S; Sakata-Sogawa K; Hasegawa A; Suzuki T; Kabu K; Sato E; Kurosaki T; Yamashita S; Tokunaga M; Nishida K; Zinc is a novel intracellular second messenger. J. Cell Biol 2007, 177, 637–645. 17502426 10.1083/jcb.200702081 PMC2064209

[62] Abdelhaleim AF; Abdo Soliman JS; Amer AY; Abdo Soliman JS Association of Zinc Deficiency with Iron Deficiency Anemia and its Symptoms: Results from a Case-control Study. Cureus 2019, 11, e3811. 30868025 10.7759/cureus.3811 PMC6402732

[63] Sanna A; Firinu D; Zavattari P; Valera P Zinc Status and Autoimmunity: A Systematic Review and Meta-Analysis. Nutrients 2018, 10, 68. 29324654 10.3390/nu10010068 PMC5793296

[64] Eide DJ Zinc transporters and the cellular trafficking of zinc. Biochim. Biophys. Acta 2006, 1763, 711–722. 16675045 10.1016/j.bbamcr.2006.03.005

[65] Lei L; Dong Z; Xu L; Yang F; Yin B; Wang Y; Yue R; Guan G; Xu J; Song G; Metal-fluorouracil networks with disruption of mitochondrion enhanced ferroptosis for synergistic immune activation. Theranostics 2022, 12, 6207–6222. 36168615 10.7150/thno.75323 PMC9475458

[66] Ohui K; Stepanenko I; Besleaga I; Babak MV; Stafi R; Darvasiova D; Giester G; Pósa V; Enyedy EA; Vegh D; Triapine Derivatives Act as Copper Delivery Vehicles to Induce Deadly Metal Overload in Cancer Cells. Biomolecules 2020, 10, 1336. 32961653 10.3390/biom10091336 PMC7564244

[67] Enyedy ÉA; Nagy NV; Zsigó É; Kowol CR; Arion VB; Keppler BK; Kiss T Comparative solution equilibrium study of the interactions of copper(II), iron(II) and zinc(II) with triapine (3-aminopyridine-2-carbaldehyde thiosemicarbazone) and related ligands. Eur. J. Inorg. Chem 2010, 2010, 1717–1728.

[68] Hager S; Pape VFS; Pósa V; Montsch B; Uhlik L; Szakács G; Tóth S; Jabronka N; Keppler BK; Kowol CR; High Copper Complex Stability and Slow Reduction Kinetics as Key Parameters for Improved Activity, Paraptosis Induction, and Impact on Drug-Resistant Cells of Anticancer Thiosemicarbazones. Antioxid. Redox Signal 2020, 33, 395–414. 32336116 10.1089/ars.2019.7854

[69] Popovic-Bijelic A; Kowol CR; Lind ME; Luo J; Himo F; Enyedy EA; Arion VB; Graslund A Ribonucleotide reductase inhibition by metal complexes of triapine (3-aminopyridine-2-carboxaldehyde thiosemicarbazone): A combined experimental and theoretical study. J. Inorg. Biochem 2011, 105, 1422–1431. 21955844 10.1016/j.jinorgbio.2011.07.003 PMC3374975

[70] Ngamchuea K; Batchelor-McAuley C; Compton RG The Copper(II)-Catalyzed Oxidation of Glutathione. Chem. Eur. J 2016, 22, 15937–15944. 27649691 10.1002/chem.201603366

[71] Chen G; Niu C; Yi J; Sun L; Cao H; Fang Y; Jin T; Li Y; Lou C; Kang J; Novel Triapine Derivative Induces Copper-Dependent Cell Death in Hematopoietic Cancers. J. Med. Chem 2019, 62, 3107–3121. 30835473 10.1021/acs.jmedchem.8b01996

[72] Gilleran JA; Yu X; Blayney AJ; Bencivenga AF; Na B; Augeri DJ; Blanden AR; Kimball SD; Loh SN; Roberge JY; Benzothiazolyl and Benzoxazolyl Hydrazones Function as Zinc Metallochaperones to Reactivate Mutant p53. J. Med. Chem 2021, 64, 2024–2045. 33538587 10.1021/acs.jmedchem.0c01360 PMC9278656

[73] Greish K Enhanced permeability and retention (EPR) effect for anticancer nanomedicine drug targeting. In Cancer Nanotechnology; Springer: Berlin/Heidelberg, Germany, 2010; pp. 25–37. 10.1007/978-1-60761-609-2_3 20217587

[74] Mitchell MJ; Billingsley MM; Haley RM; Wechsler ME; Peppas NA; Langer R Engineering precision nanoparticles for drug delivery. Nat. Rev. Drug Discov 2020, 20, 101–124. 33277608 10.1038/s41573-020-0090-8 PMC7717100

[75] Wang S; Luo J; Zhang Z; Dong D; Shen Y; Fang Y; Hu L; Liu M; Dai C; Peng S; Iron and magnetic: New research direction of the ferroptosis-based cancer therapy. Am. J. Cancer Res 2018, 8, 1933–1946. 30416846 PMC6220147

[76] Shubhra QTH Iron oxide nanoparticles in magnetic drug targeting and ferroptosis-based cancer therapy. Med. Rev 2023, 3, 444–447. 10.1515/mr-2023-0029 PMC10811351 38283254

[77] Zhang C; Liu Z; Zhang Y; Ma L; Song E; Song Y “Iron free” zinc oxide nanoparticles with ion-leaking properties disrupt intracellular ROS and iron homeostasis to induce ferroptosis. Cell Death Dis. 2020, 11, 183. 32170066 10.1038/s41419-020-2384-5 PMC7070056

[78] Yao T; Lu R; Zhang Y; Zhang Y; Zhao C; Lin R; Lin Z Cervical cancer stem cells. Cell Prolif. 2015, 48, 611–625. 26597379 10.1111/cpr.12216 PMC6495513

[79] Lee CH; Yu CC; Wang BY; Chang WW Tumorsphere as an effective in vitro platform for screening anti-cancer stem cell drugs. Oncotarget 2016, 7, 1215–1226. 26527320 10.18632/oncotarget.6261 PMC4811455

[80] Lei J-Y; Li S-X; Li F; Li H; Lei Y-S Zinc oxide nanoparticle regulates the ferroptosis, proliferation, invasion and steaminess of cervical cancer by miR-506-3p/CD164 signaling. Cancer Nanotechnol. 2022, 13, 33.

[81] Tu M; Cai L; Zheng W; Su Z; Chen Y; Qi S CD164 regulates proliferation and apoptosis by targeting PTEN in human glioma. Mol. Med. Rep 2017, 15, 1713–1721. 28259931 10.3892/mmr.2017.6204 PMC5364976

[82] Pandey NK; Chudal L; Phan J; Lin L; Johnson O; Xing M; Liu JP; Li H; Huang X; Shu Y; A facile method for the synthesis of copper–cysteamine nanoparticles and study of ROS production for cancer treatment. J. Mater. Chem. B 2019, 7, 6630–6642. 31591609 10.1039/c9tb01566c

[83] Yuan H Photothermal Nanozymatic Nanoparticles Induce Ferroptosis and Apoptosis through Tumor Microenvironment Manipulation for Cancer Therapy. Small 2022, 18, 2202161. 10.1002/smll.202202161 36089650

[84] Tong Z; Gao Y; Huang Y; Wang W; Mao Z Nanomaterials for cascade promoted catalytic cancer therapy. View 2021, 2, 20200133.

[85] Dabrowiak JC Metals in Medicine, 2nd ed.; Wiley: Hoboken, NJ, USA, 2017; pp. 91–146.

[86] Wang X; Guo Z The role of sulfur in platinum anticancer chemotherapy. Anticancer Agents Med. Chem 2007, 7, 19–34. 17266503 10.2174/187152007779314062

[87] Min Y; Mao CQ; Chen S; Ma G; Wang J; Liu Y Combating the drug resistance of cisplatin using a platinum prodrug based delivery system. Angew. Chem 2012, 51, 6742–6747. 22639083 10.1002/anie.201201562

[88] Florea A-M; Büsselberg D Cisplatin as an Anti-Tumor Drug: Cellular Mechanisms of Activity, Drug Resistance and Induced Side Effects. Cancers 2011, 3, 1351–1371. 24212665 10.3390/cancers3011351 PMC3756417

[89] Liu B; Wang H Oxaliplatin induces ferroptosis and oxidative stress in HT29 colorectal cancer cells by inhibiting the Nrf2 signaling pathway. Exp. Ther. Med 2022, 23, 394. 35495610 10.3892/etm.2022.11321 PMC9047032

[90] Imberti C; Zhang P; Huang H; Sadler PJ New Designs for Phototherapeutic Transition Metal Complexes. Angew. Chem 2020, 59, 61–73. 31310436 10.1002/anie.201905171 PMC6973108

[91] Chen G; Yang Y; Xu Q; Ling M; Lin H; Ma W; Sun R; Xu Y; Liu X; Li N; Self-Amplification of Tumor Oxidative Stress with Degradable Metallic Complexes for Synergistic Cascade Tumor Therapy. Nano Lett. 2020, 20, 8141–8150. 33172280 10.1021/acs.nanolett.0c03127

[92] Freeman EC; Weiland LM; Meng WS Modeling the proton sponge hypothesis: Examining proton sponge effectiveness for enhancing intracellular gene delivery through multiscale modeling. J. Biomater. Sci. Polym. Ed 2013, 24, 398–416. 23565683 10.1080/09205063.2012.690282 PMC3623018

[93] Sutton BM; McGusty E; Walz DT; DiMartino MJ Oral gold. Antiarthritic properties of alkylphosphinegold coordination complexes. J. Med. Chem 1972, 15, 1095–1098. 4654656 10.1021/jm00281a001

[94] Schattenkirchner M; Bröll H; Kaik B; Müller-Fassbender H; Rau R; Zeidler H Auranofin and gold sodium thiomalate in the treatment of rheumatoid arthritis: A one-year, double-blind, comparative multicenter study. Klin. Wochenschr 1988, 66, 167–174. 3131571 10.1007/BF01727786

[95] Simon TM; Kunishima DH; Vibert GJ; Lorber A Cellular antiproliferative action exerted by auranofin. J. Rheumatol. Suppl 1979, 5, 91–97. 226702

[96] Allardyce CS; Dyson PJ Metal-based drugs that break the rules. Dalton Trans. 2016, 45, 3201–3209. 26820398 10.1039/c5dt03919c

[97] Fernández-Vega L; Ruiz Silva VA; Domínguez-González TM; Claudio-Betancourt S; Toro-Maldonado RE; Capre Maso LC; Sanabria Ortiz K; Pérez-Verdejo JA; Román González J; Rosado-Fraticelli GT; Evaluating ligand modifications of the titanocene and auranofin moieties for the development of more potent anticancer drugs. Inorganics 2020, 8, 10. 34046448 10.3390/inorganics8020010 PMC8152503

[98] Lippmann J Redox Modulation and Induction of Ferroptosis as a New Therapeutic Strategy in Hepatocellular Carcinoma. Transl. Oncol 2020, 13, 100785. 32416440 10.1016/j.tranon.2020.100785 PMC7283515

[99] Nobili S; Mini E; Landini I; Gabbiani C; Casini A; Messori L Gold compounds as anticancer agents: Chemistry, cellular pharmacology, and preclinical studies. Med. Res. Rev 2010, 30, 550–580. 19634148 10.1002/med.20168

[100] Rigobello MP; Scutari G; Folda A; Bindoli A Mitochondrial thioredoxin reductase inhibition by gold(I) compounds and concurrent stimulation of permeability transition and release of cytochrome c. Biochem. Pharmacol 2004, 67, 689–696. 14757168 10.1016/j.bcp.2003.09.038

[101] Freire Boullosa L; Van Loenhout J; Flieswasser T; De Waele J; Hermans C; Lambrechts H; Cuypers B; Laukens K; Bartholomeus E; Siozopoulou V; Auranofin reveals therapeutic anticancer potential by triggering distinct molecular cell death mechanisms and innate immunity in mutant p53 non-small cell lung cancer. Redox Biol. 2021, 42, 101949. 33812801 10.1016/j.redox.2021.101949 PMC8113045

[102] Ye S; Chen X; Yao Y; Li Y; Sun R; Zeng H; Shu Y; Yin H Thioredoxin Reductase as a Novel and Efficient Plasma Biomarker for the Detection of Non-Small Cell Lung Cancer: A Large-scale, Multicenter study. Sci. Rep 2019, 9, 2652. 30804354 10.1038/s41598-018-38153-7 PMC6389956

[103] Karlenius TC; Tonissen KF Thioredoxin and Cancer: A Role for Thioredoxin in all States of Tumor Oxygenation. Cancers 2010, 2, 209–232. 24281068 10.3390/cancers2020209 PMC3835076

[104] Kalo E; Kogan-Sakin I; Solomon H; Bar-Nathan E; Shay M; Shetzer Y; Dekel E; Goldfinger N; Buganim Y; Stambolsky P; Mutant p53R273H attenuates the expression of phase 2 detoxifying enzymes and promotes the survival of cells with high levels of reactive oxygen species. J. Cell Sci 2012, 125, 5578–5586. 22899716 10.1242/jcs.106815

[105] Monro S; Colón KL; Yin H; Roque J 3rd; Konda P; Gujar S; Thummel RP; Lilge L; Cameron CG; McFarland SA Transition Metal Complexes and Photodynamic Therapy from a Tumor-Centered Approach: Challenges, Opportunities, and Highlights from the Development of TLD1433. Chem. Rev 2019, 119, 797–828. 30295467 10.1021/acs.chemrev.8b00211 PMC6453754

[106] Tian J; Huang B; Nawaz MH; Zhang W Recent advances of multi-dimensional porphyrin-based functional materials in photodynamic therapy. Coord. Chem. Rev 2020, 420, 213410.

[107] Yuan H; Han Z; Chen Y; Qi F; Fang H; Guo Z; Zhang S; He W Ferroptosis Photoinduced by New Cyclometalated Iridium(III) Complexes and Its Synergism with Apoptosis in Tumor Cell Inhibition. Angew. Chem 2021, 60, 8174–8181. 33656228 10.1002/anie.202014959

[108] Lu N; Deng Z; Gao J; Liang C; Xia H; Zhang P An osmium-peroxo complex for photoactive therapy of hypoxic tumors. Nat. Commun 2022, 13, 2245. 35473926 10.1038/s41467-022-29969-z PMC9042834

